# Somatic mutations in single human cardiomyocytes reveal age-associated DNA damage and widespread oxidative genotoxicity

**DOI:** 10.1038/s43587-022-00261-5

**Published:** 2022-08-11

**Authors:** Sangita Choudhury, August Yue Huang, Junho Kim, Zinan Zhou, Katherine Morillo, Eduardo A. Maury, Jessica W. Tsai, Michael B. Miller, Michael A. Lodato, Sarah Araten, Nazia Hilal, Eunjung Alice Lee, Ming Hui Chen, Christopher A. Walsh

**Affiliations:** 1 Division of Genetics and Genomics, Manton Center for Orphan Disease, Department of Pediatrics, Boston Children’s Hospital, Boston, MA, USA.; 2 Departments of Pediatrics, Harvard Medical School, Boston, MA, USA.; 3 Broad Institute of MIT and Harvard, Cambridge, MA, USA.; 4 Department of Biological Sciences, Sungkyunkwan University, Suwon, South Korea.; 5 Department of Neurology, Harvard Medical School, Boston, MA, USA.; 6 Bioinformatics & Integrative Genomics Program and Harvard/MIT MD-PhD Program, Harvard Medical School, Boston, MA, USA.; 7 Department of Pediatric Oncology, Dana-Farber Cancer Institute, Boston, MA, USA.; 8 Division of Neuropathology, Department of Pathology, Brigham and Women’s Hospital, Harvard Medical School, Boston, MA, USA.; 9 Department of Molecular, Cell and Cancer Biology, University of Massachusetts Medical, School, Worcester, MA, USA.; 10 Department of Cardiology, Boston Children’s Hospital, Boston, MA, USA.; 11 Harvard Medical School, Boston, MA, USA.; 12 Howard Hughes Medical Institute, Boston Children’s Hospital, Boston, MA, USA.; 13 These authors contributed equally: Sangita Choudhury, August Yue Huang.; 14 These authors jointly supervised this work: Sangita Choudhury, Eunjung Alice Lee, Ming Hui Chen, Christopher A. Walsh.

## Abstract

The accumulation of somatic DNA mutations over time is a hallmark of aging in many dividing and nondividing cells but has not been studied in postmitotic human cardiomyocytes. Using single-cell whole-genome sequencing, we identified and characterized the landscape of somatic single-nucleotide variants (sSNVs) in 56 single cardiomyocytes from 12 individuals (aged from 0.4 to 82 years). Cardiomyocyte sSNVs accumulate with age at rates that are faster than in many dividing cell types and nondividing neurons. Cardiomyocyte sSNVs show distinctive mutational signatures that implicate failed nucleotide excision repair and base excision repair of oxidative DNA damage, and defective mismatch repair. Since age-accumulated sSNVs create many damaging mutations that disrupt gene functions, polyploidization in cardiomyocytes may provide a mechanism of genetic compensation to minimize the complete knockout of essential genes during aging. Age-related accumulation of cardiac mutations provides a paradigm to understand the influence of aging on cardiac dysfunction.

Advanced age is the most critical risk factor for heart disease and heart failure^1^. Most heart failure occurs in individuals older than 65 years, and yet we have an incomplete understanding of how aging promotes heart failure. With aging, cardiac function is impaired at the organ level as well as at the cardiomyocyte level^2^. In the adult heart, cardiomyocyte cell size is stable, and proliferation is either very rare or absent. Nevertheless, cardiomyocytes show changes in activation, contraction and relaxation, hypertrophy and, in some cases, cell loss with aging^3^. The underlying cause of these many aging phenotypes is likely molecular in nature, but its mechanism is not well understood.

Somatic mutations are genomic changes that escape the DNA repair machinery, and occur after the formation of the zygote and throughout the whole lifespan. The accumulation of somatic DNA mutations has been demonstrated to be a hallmark of aging in many human cell types, including postmitotic neurons^4–7^. In most cases, they have little or no phenotypic consequences, as most mutations have no effect on cellular function. In some cases, these mutations are toxic, or even lethal, to the mutant cell. Recent evidence suggests that somatic mutations play a role in cancer as well as in other common diseases^8–10^, including coronary artery disease^11^. Each mutational process leaves a characteristic mutational mark in the genomic context (so-called mutational signature) and many of these mutational signatures have been linked to specific mutagen and biological processes, such as tobacco smoke or the deficiency of DNA repair processes^12^. Understanding mutational signatures and their mechanism of formation might lead us to unveil the mechanism of DNA damage and disease progression in the aging heart. This prompted us to evaluate the landscape of somatic single-nucleotide variants (sSNVs) and associated mutational signatures in aging human cardiomyocytes.

## Results

### Cardiomyocyte polyploidization starts early in life.

**Extended Data Fig. 1 |:**
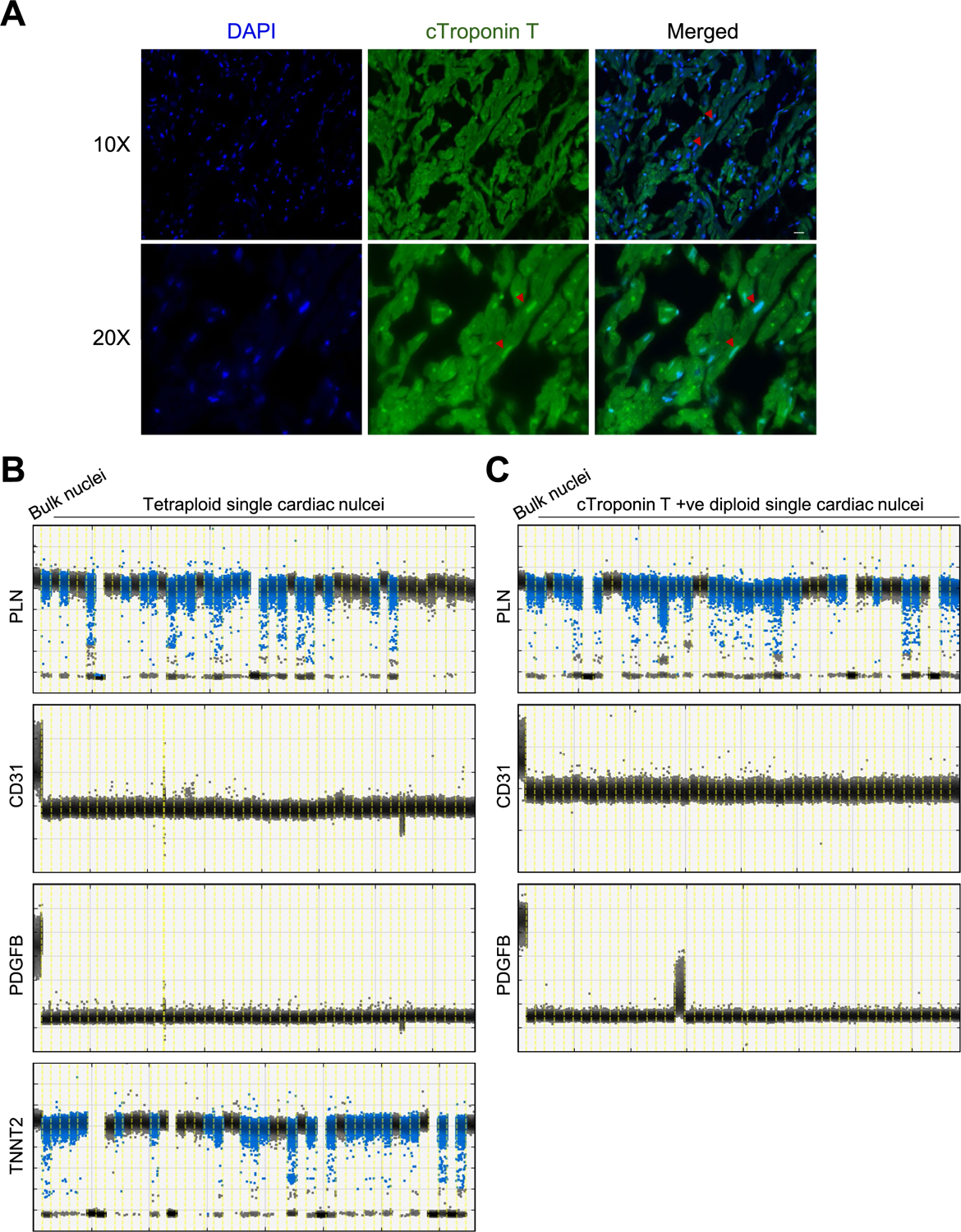
isolation and identification of cardiac nuclei. **A**, Representative immune-histochemical detection of cTroponin T in cardiomyocyte nuclei. Section from non-diseased human left ventricle tissue stained against cTroponin T and the nuclear marker DAPI demonstrating nuclear labeling in cardiomyocytes. Scale bar, 20 μm. **B**,**C**, Purity check of fluorescence-activated sorted cardiomyocyte nuclei. ddPCR analysis (n = 4 experiments for 12 cases) of tetraploid cardiac nuclei (**B**), and cTroponin T+ve diploid cardiac nuclei (**C**). Both tetraploid and cTroponin T positive diploid cardiomyocytes are highly enriched for *PLN* and *TNNT2*, cardiac markers, but not *PDGFB* or *CD31*, markers for fibroblast and endothelial cells. Each lane represents 1 single nucleus, except the first lane, containing 100 nuclei. The bottom clusters on the plot represent the negative droplets and the upper clusters represent the droplets that are positive for the respective reference assay.

**Fig. 1 |:**
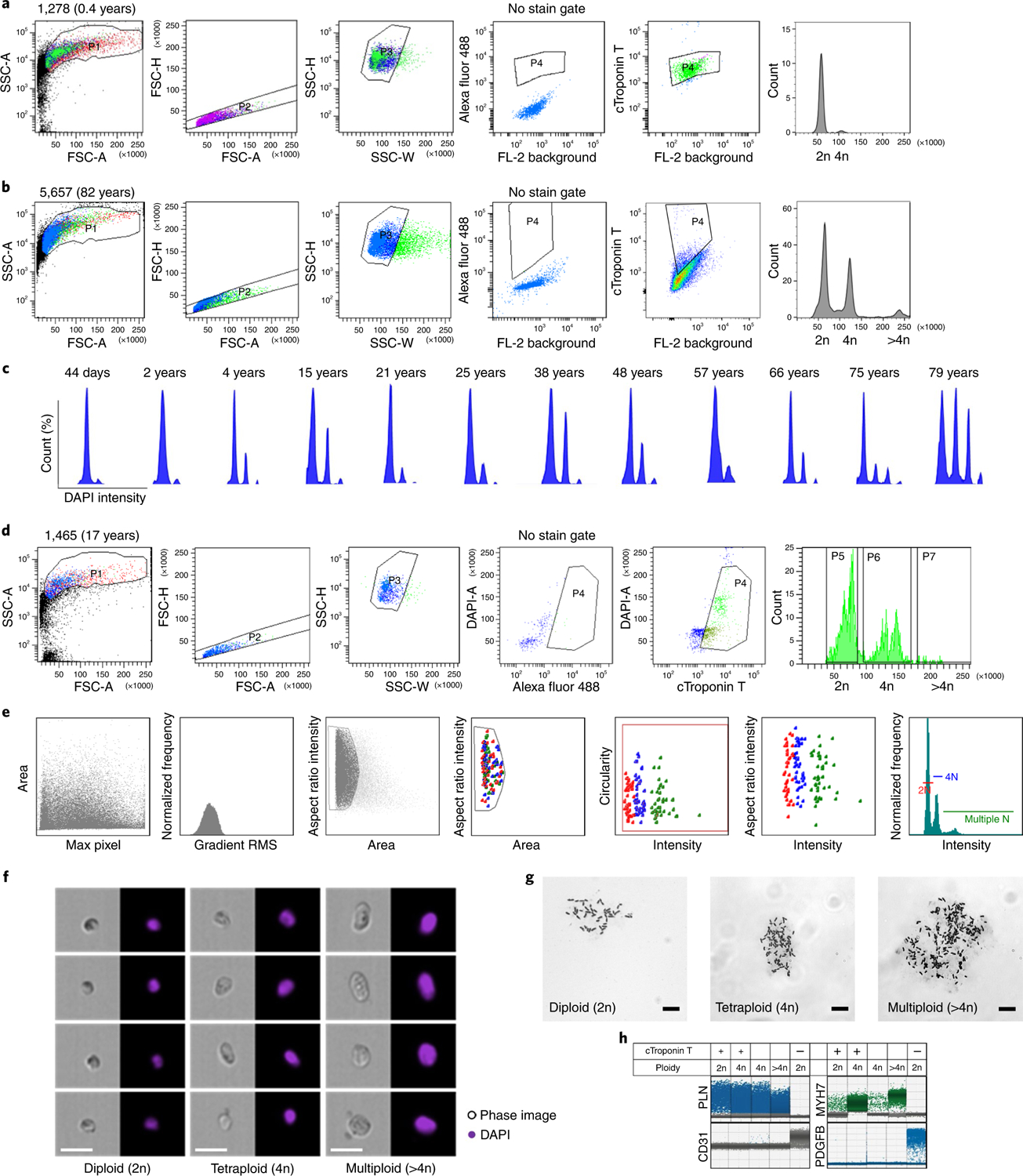
Evaluating cardiomyocyte nuclei ploidy in postmortem human heart. **a**,**b**,**d**, Representative flow cytometry analysis of cardiac nuclei with Alexa Fluor 488 conjugate cTroponin T with careful doublet exclusion for 1278 (**a**), 5657 (**b**), and 1465 (**d**). **c**, DNA content histograms of heart cell nuclei with varied ages. The first peak indicates diploid (2n) nuclei, the second peak indicates tetraploid (4n) nuclei and the third and fourth peaks indicate multiploid (>4n) nuclei. **d**,**e**, Evaluation of cardiomyocyte nuclei ploidy by flow cytometry (**d**) and Amnis imaging flow cytometry (**e**), showing diploid (2N), tetraploid (4N) and multiploid (multiple N) cardiomyocyte nuclei proportion in case 1465. **f**, Representative photomicrographs of isolated cardiomyocyte nuclei (*n* = 4 independent experiments; images from 500 cells per experiment were examined from *n* = 20 cases), confirming DNA content of a single tetraploid and multiploid cardiomyocyte nuclei. Scale bar, 20 μm. **g**, Flow cytometry sorted cardiomyocyte nuclei karyotyping confirming cardiomyocyte chromosomes numbers, 46 (2n), 92 (4n) and 138 (>4n) in cardiomyocyte nuclei isolated from case 1465 (*n* = 6; 15–20 nuclei counted each time). Scale bar, 10 μm. **h**, Representative ddPCR analysis from flow cytometry sorted nuclei (*n* = 4, from 12 cases). cTroponin-T-positive 2n, 4n and greater than 4n are highly enriched for *PLN* and *MYH7* (cardiac markers), whereas cTroponin-T-negative 2n nuclei express *PDGFB* or *CD31* (markers for fibroblast and endothelial cells). The bottom clusters on the plot represent the negative droplets and the upper clusters represent the droplets that are positive for the respective reference assay. The plus sign indicates cells sorted from cTroponin-T-positive staining population; the dash indicates cells sorted from cTroponin-T-negative population; the blank box indicates that cells were sorted based solely on ploidy status

To identify and isolate cardiomyocyte nuclei from heart tissue, we purified cardiac nuclei from fresh-frozen, unfixed, left ventricle human myocardium by density sedimentation (Supplementary Table 1)^13^. Cardiac nuclei were identified with cardiac troponin T (cTroponin T) staining (Fig. 1a,b,d and Extended Data Fig. 1a), which is a well-characterized cardiomyocyte-specific marker^14^. Although the pericentriolar material 1 (PCM1) antibody is used widely to identify cardiomyocyte nuclei, it has been shown that about 20% of PCM1-positive nuclei correspond to nonmyocyte cells^15^. Careful doublet exclusion was performed by plotting the area for forward scatter (FSC) and side scatter (SSC) against the height (H) or width (W); H versus W or area (A) allows the separation of doublets from single nuclei containing 4n amounts of DNA. Nuclei containing 4n amounts of DNA have double the A and H values, whereas W is roughly the same as cells containing 2n amounts of DNA (Fig. 1a,b; DAPI-H versus DAPI-W). We evaluated systematically cardiomyocyte nucleus ploidy from 50 human heart tissue samples from individuals ranging from 44 days to 81 years of age (Fig. 1c, showing only representative age group plots). Polyploid cardiomyocyte nuclei were identified based on the intensity of nuclear stain 4′,6-diamidino-2-phenylindole (DAPI). Fluorescence-activated nuclei sorting (FANS Fig. 1c,d), as well as Amnis imaging flow (Fig. 1e,f), revealed the presence of modest numbers of tetraploid cardiomyocyte nuclei even in the newborn heart tissue. Cardiomyocyte ploidy was further confirmed after nuclear sorting with karyotyping (Fig. 1g). Single-cell gene expression analysis by droplet digital PCR (ddPCR) from tetraploid nuclei indicates that tetraploid nuclei all express cardiac marker genes *PLN*, *TNNT2* and *MYH7* and lack fibroblast and endothelial marker genes (Fig. 1h and Extended Data Fig. 1b), suggesting that all tetraploid nuclei are cardiomyocytes, and making it unlikely that tetraploid nuclei represent sorting artifacts. Extensive studies by fluorescence in situ hybridization and immunostaining have indicated that human cardiomyocyte nuclei are mostly diploid at birth and start to become polyploid mainly in the second decade of life^16^, and that 60% of human cardiomyocyte nuclei have higher ploidy at an advanced age^17–19^. Our data indicate that polyploidization in cardiomyocytes is evident in the neonatal heart but confirm that it becomes more common with age.

### Somatic mutations increase with age in cardiomyocytes.

**Fig. 2 |:**
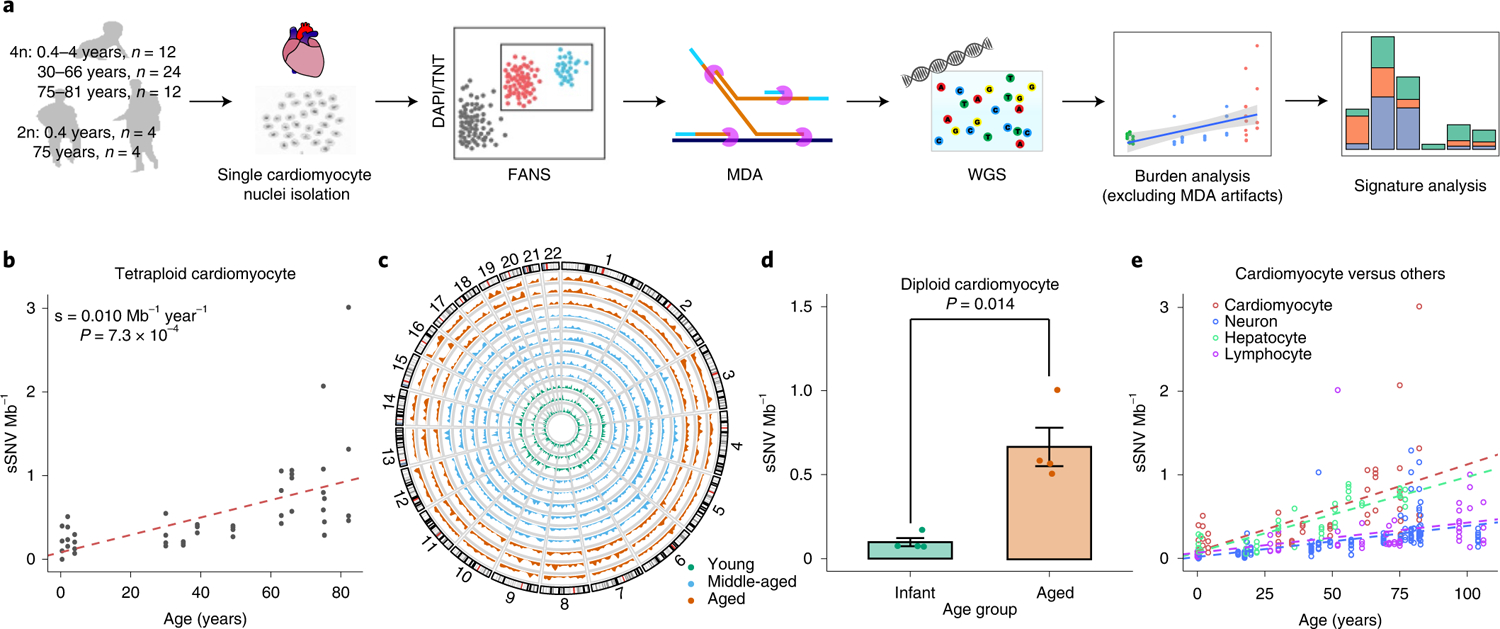
sSNV density in single human cardiomyocytes detected by single-cell WgS. **a**, Schematic of approach. Nuclei are isolated from frozen postmortem heart tissues with varied ages, and sorted based on ploidy content by FANS. Sorted nuclei are amplified by **Φ**29 polymerase-mediated MDA for WGS and subsequent sSNV burden and signature analysis. The numbers of examined nuclei (*n*) are noted. **b**, Estimated sSNV density in tetraploid cardiomyocytes. Tetraploid cardiomyocytes showed an increased number of sSNVs with increased age (*P* = 7.3 × 10^−4^, mixed-effects regression). Single tetraploid cardiac nuclei were obtained from normal postmortem left ventricle hearts of individuals ranging from 0.4 to 82.7 years of age (*n* = 48 cells from 12 donors). **c**, Genome-wide distribution of SNVs. Autosomal sSNV density in the 10 Mb genomic window is plotted for each individual. Green, blue and red curves represent infant, middle-aged and aged hearts, respectively. **d**, Estimated sSNV density in diploid cardiomyocytes. Aged diploid cardiomyocytes (75 years, *n* = 4 cells) showed an increase of around sevenfold (*P* = 0.014, two-tailed *t*-test) in sSNV burden compared with infant cardiomyocytes (0.4 years, *n* = 4 cells). Error bar, ± s.e.m. **e**, Age-dependent increases of sSNV density in cardiomyocytes versus neurons, hepatocytes and lymphocytes. Red, blue, green and purple dotted lines indicate the mixed-effects regression lines of cardiomyocytes, neurons, hepatocytes and lymphocytes, respectively.

We evaluated the genome-wide burden of sSNVs using single-cell whole-genome sequencing (WGS) of DNA amplified from 48 tetraploid and 8 diploid single-cardiomyocyte nuclei from postmortem hearts of three infant (<4 years), six middle-aged (30–66 years) and three aged individuals (>75 years) (Fig. 2a and Table 1). Cardiomyocyte nuclei were isolated from the left ventricle, and DNA was amplified using multiple displacement amplification (MDA)^20^ followed by quality control steps (Methods) and then high-coverage (>50×) WGS on selected, well-amplified cells^10^ (Supplementary Table 2). We identified single-cell sSNVs from each cardiomyocyte (Supplementary Table 3) using a modified version of the LiRA^21^ algorithm, which uses read phasing information from adjacent germline variants to distinguish true somatic mutations from technical artifacts arising during whole-genome amplification and sequencing. The LiRA algorithm has been demonstrated to effectively remove most false positives^21^. We also estimated the genome-wide sSNV burden for each cardiomyocyte (Supplementary Table 4), after taking cell-specific dropout rates and sequencing depth distributions into consideration to account for the tetraploidy effect on detection sensitivity and power calculation, and subtracting the contribution of potential artifacts due to MDA amplification based on their highly distinctive nucleotide substitution pattern^22,23^ (Methods). We also measured the MDA amplification evenness across the genome for each single-cell using median absolute pairwise difference (MAPD) and coefficient of variation (CoV) of binned normalized copy number ratios, and incorporated them as covariates in our subsequent regression analyses.

**Extended Data Fig. 2 |:**
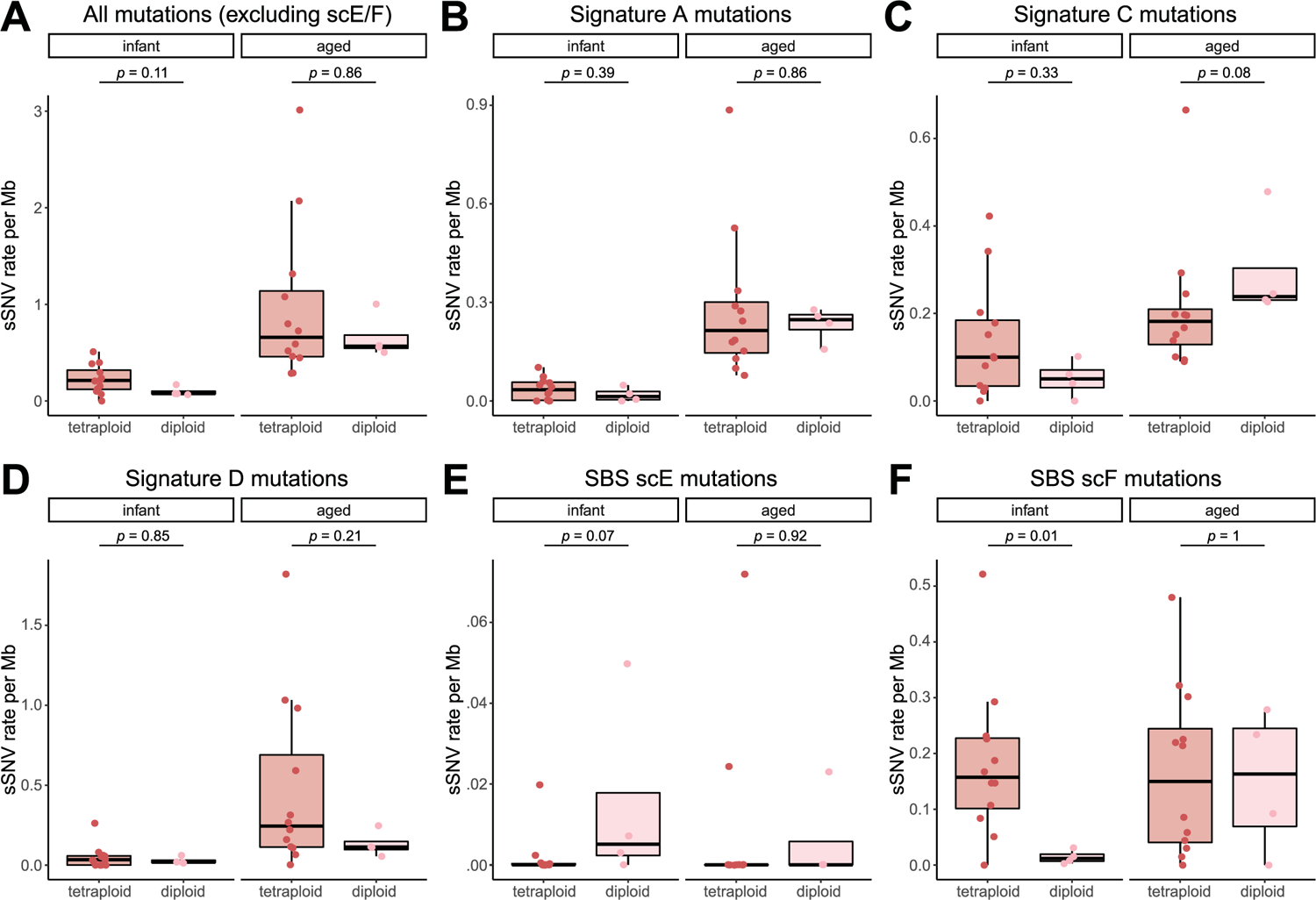
Comparison of sSNV burden between tetraploid and diploid cardiomyocytes for total mutations **(A) and per-signature mutations (B–F).** Except for the SBS scF mutations in infant cardiomyocytes, there were no statistically significant differences (*p* > 0.05, two-tailed Wilcoxon test) in the mutation burden between tetraploid (n = 12 for infant and aged each) and diploid (n = 4 for infant and aged each) cardiomyocytes. Boxplot with whisker denotes minimum, 25%, median, 75% quartiles, and maximum.

Tetraploid cardiomyocytes showed a significant increase of sSNV with age (*P* = 7.3 × 10^−4^, mixed-effects regression; Fig. 2b) at a rate of 0.010 sSNV Mb^−1^ year^−1^ (or 124 sSNV cell^−1^ year^−1^), and the sSNVs were distributed broadly across the genome (Fig. 2c). Note that, in each age group, there are notable intraindividual and interindividual variations, particularly in the aged group, where a few outlier nuclei showed a very high sSNV burden (>2 sSNV Mb^−1^). We further confirmed the age-dependent increase in diploid cardiomyocytes, in which the aged heart showed significantly more sSNVs than the infant heart (*P* = 0.014, two-tailed *t*-test; Fig. 2d). We observed nearly doubled per-genome sSNV rate in tetraploid cardiomyocytes when compared with diploid cardiomyocytes, but, after normalizing to their different genomic size, no significant difference was observed between tetraploid and diploid cardiomyocytes obtained from infant and aged donors (*P* = 0.11 and 0.86, two-tailed Wilcoxon test; Extended Data Fig. 2a), suggesting a consistent increase of sSNV in human heart muscle cells with age, regardless of cardiomyocyte nuclear ploidy. Since two of our donors were affected by ventricular hypertrophy (cases 5657 and 5840), we remeasured the age-associated increase after excluding cardiomyocytes obtained from these two donors, and still observed a consistent increase rate (0.010 sSNV Mb^−1^ year^−1^; *P* = 6.1 × 10^−4^, mixed-effects regression).

**Extended Data Fig. 3 |:**
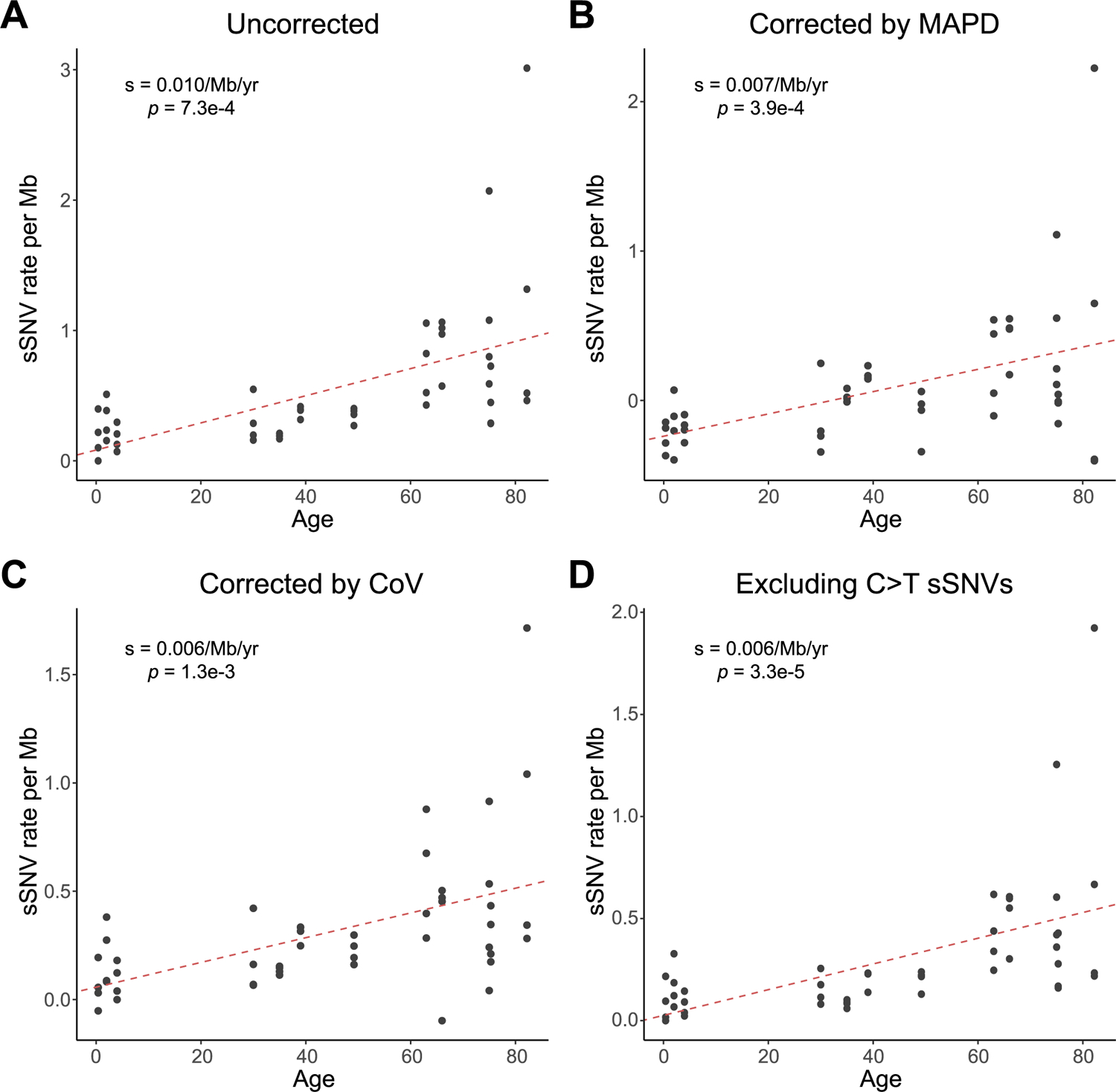
Evaluation of sSNV burden in cardiomyocytes with the consideration of amplification evenness or non-C > T mutations. **A**, Uncorrected sSNV density of tetraploid cardiomyocytes. **B**–**C**, sSNV density of tetraploid cardiomyocytes after correcting for two metrics about amplification evenness, MAPD (**B**) and CoV (**C**). **D**, sSNV density of tetraploid cardiomyocytes after excluding C > T mutations. Tetraploid cardiomyocytes showed consistent age-dependent accumulation of sSNVs, robust to amplification evenness (*p* = 3.9 × 10^−4^ for MAPD correction and 1.3 × 10^−3^ for CoV correction, mixed-effects model) and the exclusion of C > T mutations (*p* = 3.3 × 10^−5^, mixed-effects model).

To better understand the age-dependent sSNV accumulation in cardiomyocytes, we first compared the accumulation rates between cardiomyocytes and neurons^10,23^, another nondividing human cell type. We found that cardiomyocytes accumulate sSNVs around three times faster than neurons (0.003 sSNV Mb^−1^ year^−1^ or 19 sSNV cell genome^−1^ year^−1^; *P* = 2.1 × 10^−5^ between cardiomyocyte and neuron, mixed-effects regression; Fig. 2e), suggesting that cardiomyocytes and neurons might undergo different mutational processes during aging. The age-dependent increase in cardiomyocytes and the cardiomyocyte-neuron difference remained significant (*P* < 0.05) even after controlling potential confounding factors including MAPD and CoV scores (measurement of amplification evenness), sequencing depth, library insert size, number of heterozygous germline SNVs and postmortem interval (PMI), as well as excluding C > T sSNVs (Extended Data Fig. 3; Methods). We further compared the sSNV accumulation rate in cardiomyocytes with hepatocytes^24^—the liver cells of another highly active metabolic organ—and mitotically active lymphocyte blood cells^25^ profiled by other groups (Fig. 2e). We found that cardiomyocytes accumulated sSNV at a rate similar to that of hepatocytes (0.009 sSNV Mb^−1^ year^−1^ considering diploid genome or 55 sSNV cell^−1^ year^−1^; *P* = 0.54 between cardiomyocyte and hepatocyte, mixed-effects regression) but significantly higher than lymphocytes (0.004 sSNV Mb^−1^ year^−1^ or 22 sSNV cell^−1^ year^−1^; *P* = 2.0 × 10^−4^ between cardiomyocyte and lymphocyte, mixed-effects regression).

### Signature analysis identifies distinct mutational processes in aging cardiomyocytes.

**Extended Data Fig. 4 |:**
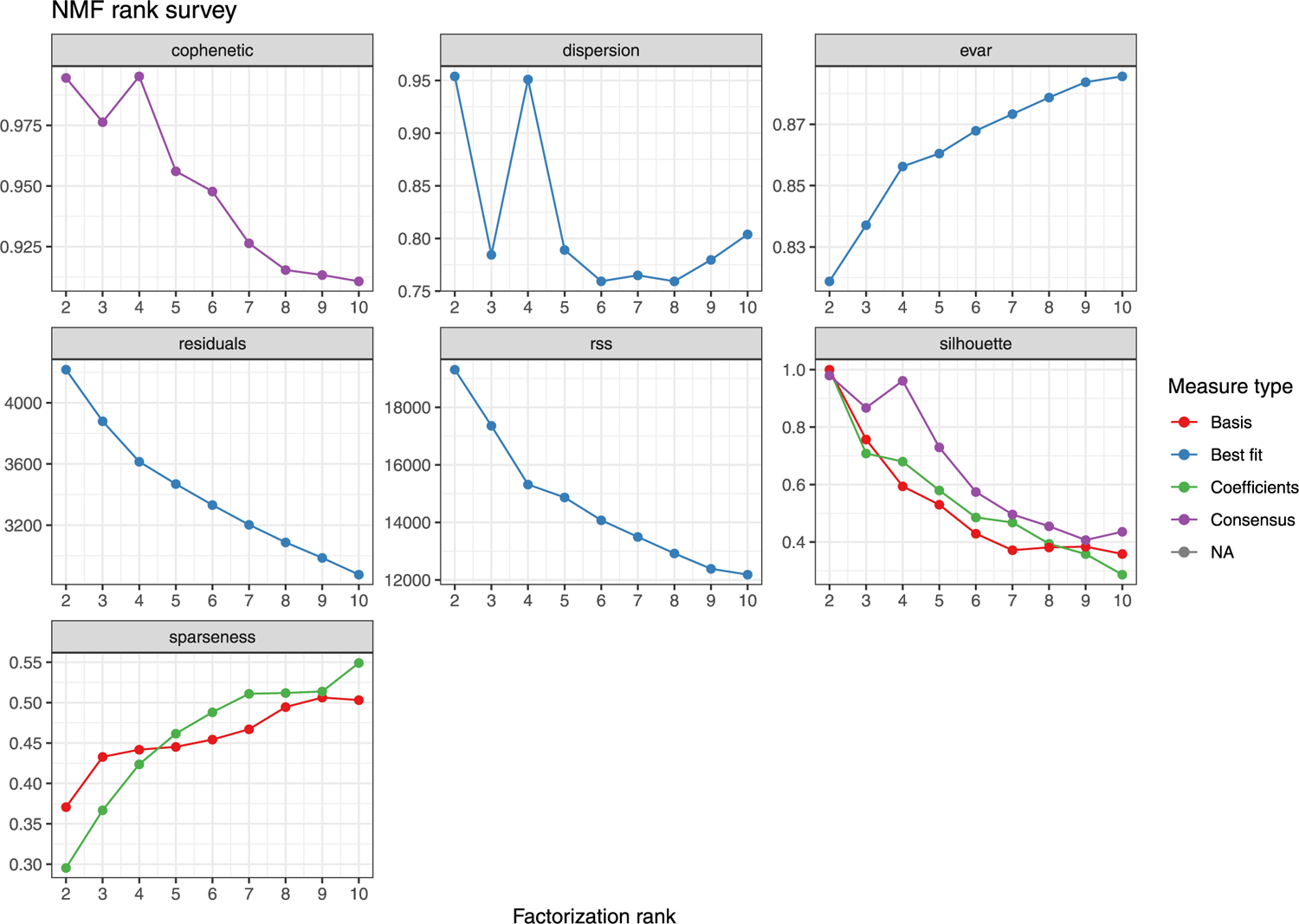
Signature metrics for de novo mutational signature analysis. *De novo* mutational signature analysis was performed using non-negative matrix factorization (NMF), in which the factorization rank is critical to define the number of signatures used to decompose the target matrix of sSNVs. We identified that four signatures can maximize the cophenetic and best fit the observed sSNV matrix.

**Extended Data Fig. 5 |:**
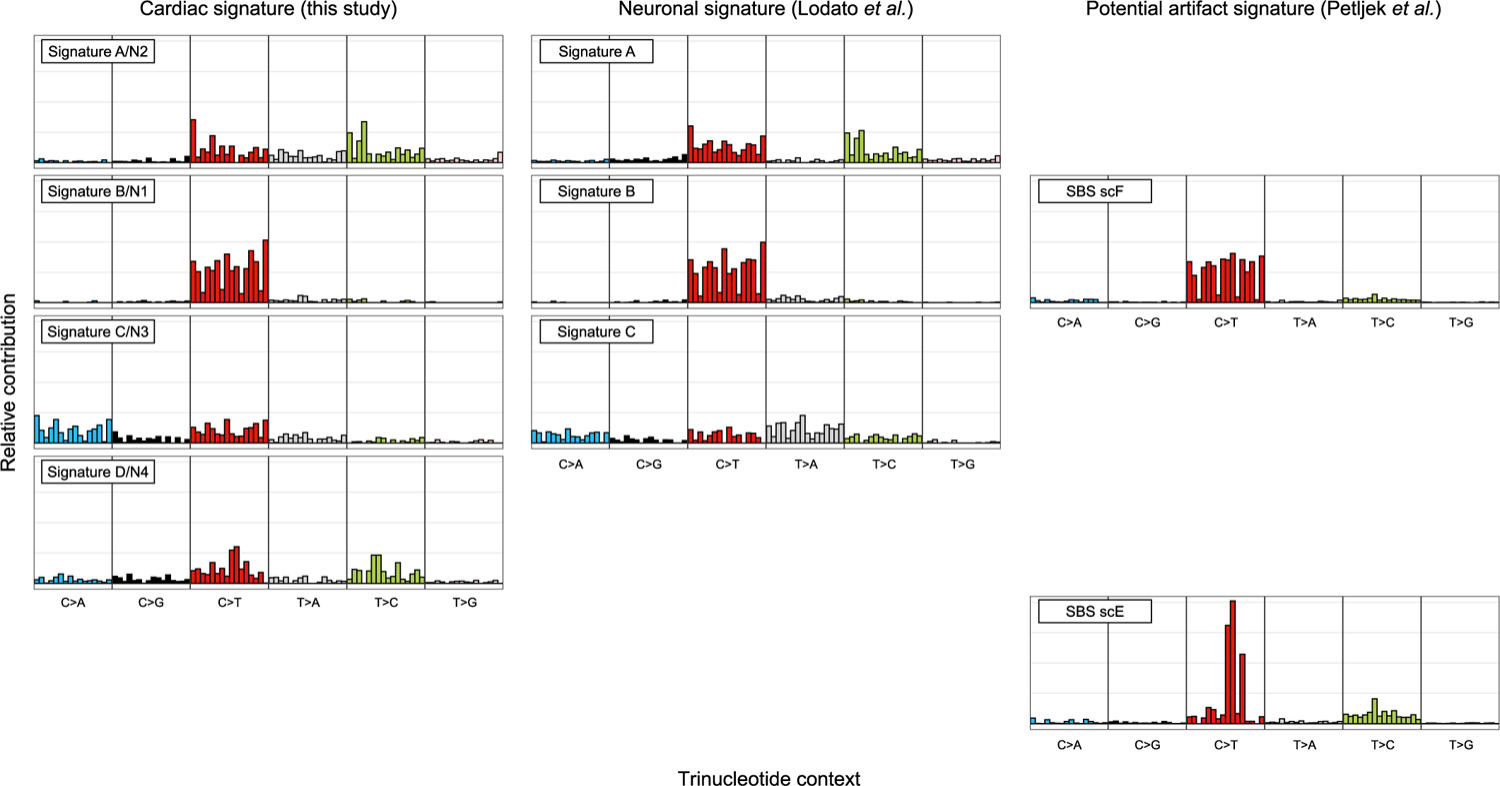
Comparison of mutational signatures identified in this and other studies. (Left panel) *De novo* mutational signatures identified from single human cardiomyocytes in this study. (Middle panel) Previously published signatures identified from single human neurons (Lodato et al.). (Right panel) Recently published signatures thought to represent artifacts of single-cell whole genome amplification, SBS scE and scF, from a study of cultured cells (Petljak et al.). Signature D/N4 was present only in cardiomyocytes but not in neurons. Signature B/N1 identified in cardiomyocytes and neurons resembles the artifact signature SBS scF, thus it was excluded for further mutational burden and signature analyses in this study.

**Fig. 3 |:**
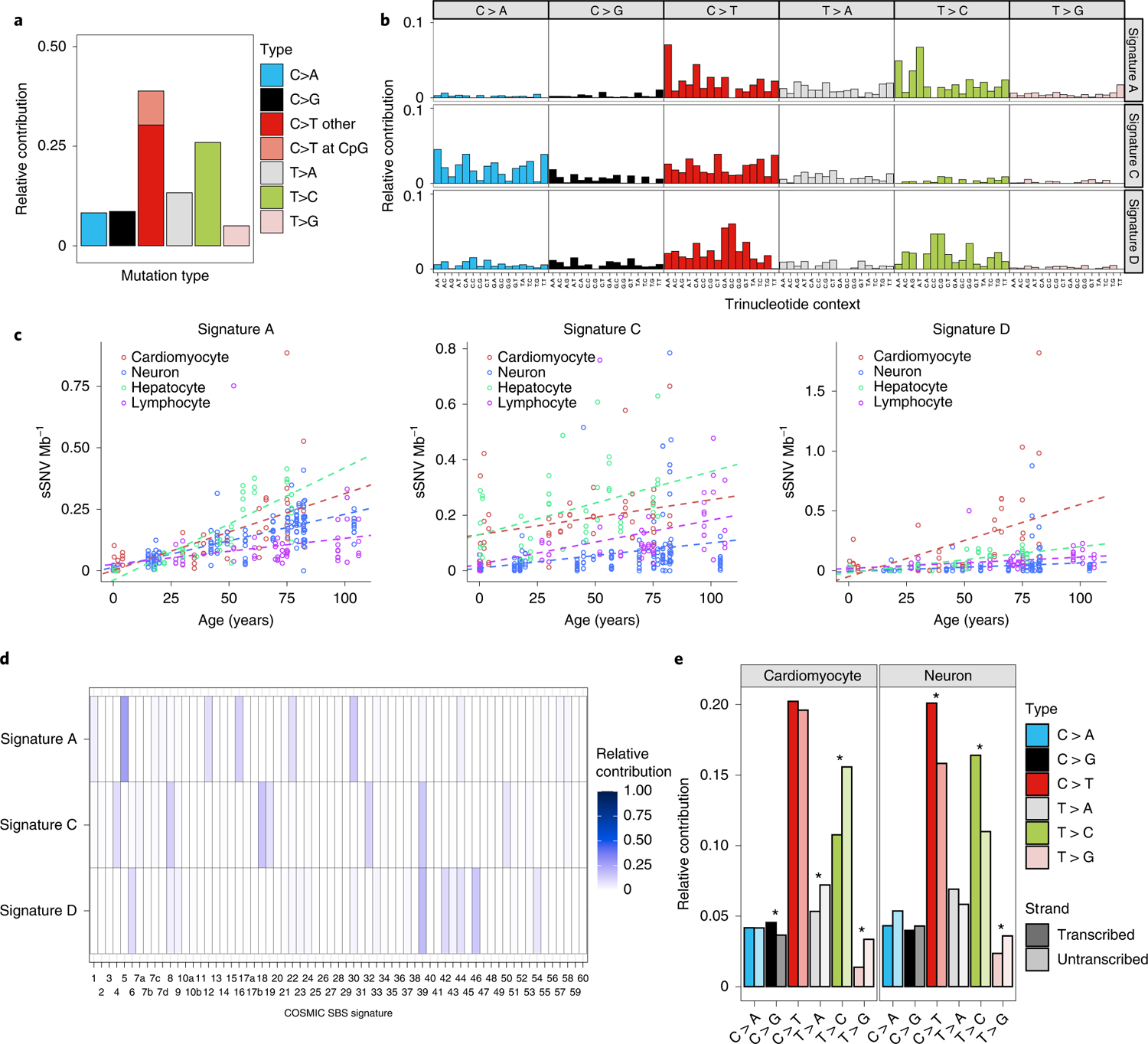
Signature analysis of sSNV reveals mutational processes in cardiomyocytes during aging. **a**, Substitution type for the age-accumulated sSNVs (the ‘net increase’ in sSNVs between infant and aged cardiomyocytes). C > T and T > C substitutions are predominant. **b**, Cardiac mutational signatures identified by NMF based on the trinucleotide context of sSNVs. Each signature is displayed according to the 96 trinucleotide contexts, defined by the six substitution types and sequence context immediately 5′ and 3′ to the mutated base. Although both Signatures A and D predominate with C > T and T > C substitutions, they differ in trinucleotide contexts at C > T and T > C substitutions. **c**, Signature-specific sSNV density in cardiomyocytes, neurons, hepatocytes and lymphocytes. Signature D specifically accumulated in aged cardiomyocytes, whereas Signature A accumulated with age in all cell types but at different rates. **d**, NMF-based decomposition of cardiac signatures into COSMIC cancer signatures. The relative contribution of each cancer signature is shown as heatmaps. **e**, Transcription bias of age-accumulated sSNVs. Asterisks mark significant difference between transcribed and untranscribed strands (two-tailed Poisson test) in cardiomyocytes (C > G, *P* = 4.7 × 10^−3^; T > A, *P* = 1.2 × 10^−6^; T > C, *P* < 2.2 × 10^−16^; T > G, *P* < 2.2 × 10^−16^) and in neurons (C > T, *P* = 7.3 × 10^−4^; T > C, *P* = 8.9 × 10^−7^; T > G, *P* = 1.6 × 10^−2^). Notably, T > C substitution is enriched in the untranscribed strand in cardiomyocytes, whereas it is enriched in the transcribed strand in neurons, suggesting different mutation mechanisms in these two nondividing cells.

Different types of mutagenesis processes manifest different mutational signatures on the genome, offering insight into the molecular mechanisms involved in their formation^26^. Using 10,407 sSNVs identified from 48 tetraploid cardiac nuclei (Supplementary Table 3), we first studied the base substitution distribution of age-accumulated sSNVs by subtracting the sSNV profiles of infant cardiomyocytes from those of aged cardiomyocytes, and observed that C > T and T > C mutations accumulated predominantly during the aging process (Fig. 3a). To further decipher mutational processes in the aging heart, we deconvoluted the sSNV profiles of all cardiomyocytes into mutation signatures using non-negative matrix factorization (NMF)^27^. Cardiac sSNVs were best fit into four distinct mutational signatures. referred to in this study as Signature A, B, C and D (Extended Data Fig. 4 and Extended Data Fig. 5). Signature B consisted mainly of C > T mutations, depleted at CpG dinucleotides, and matched closely with mutational signatures that were previously ascribed to artifacts created by MDA amplification^22^, so Signature B was removed from mutation burden analysis and not considered further. We compared our cardiac signatures with single-base substitution (SBS) signatures annotated by the COSMIC (Catalogue Of Somatic Mutations In Cancer) database^28^, a curated list of reference signatures generated from distinct types of human cancer, many of which had linked their mutagenesis mechanisms to various environmental factors and intrinsic processes. Further, we confirmed that tetraploid and diploid cardiomyocytes showed a similar contribution of Signatures A, C and D (Extended Data Fig. 2b,c,d).

**Extended Data Fig. 6 |:**
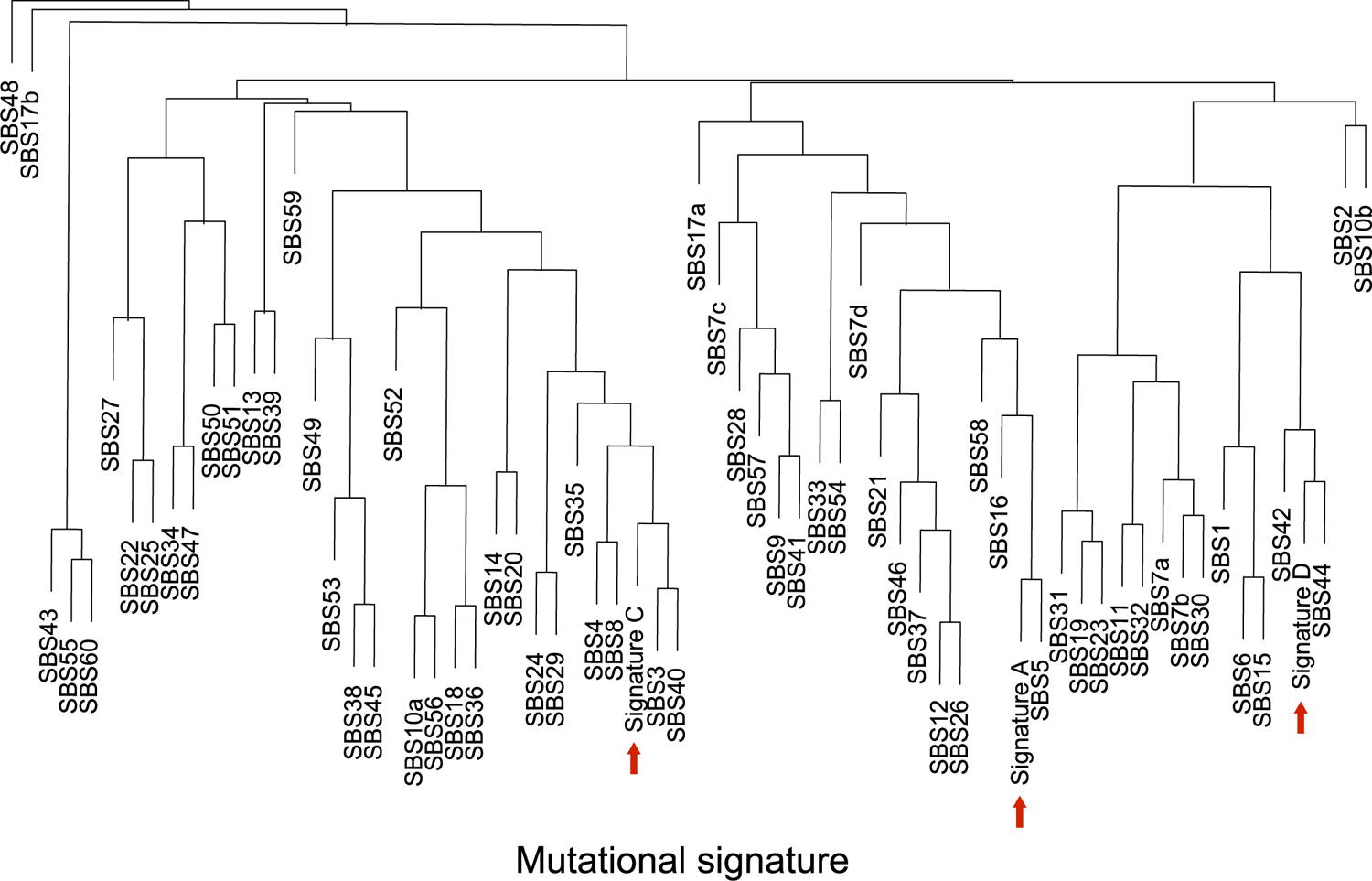
Hierarchical clustering between cardiac and COSMiC cancer signatures. Unsupervised clustering was performed among single-cardiomyocyte-derived signatures (Signatures A, C, and D) and cancer-derived COSMIC signatures (SBS1–60), by using cosine similarity of 96-class of trinucleotide context to measure the pairwise distance. Signature A resembles SBS5 and Signature D resembles SBS44.

Signature A comprised mainly C > T and T > C mutations (Fig. 3b), and its contribution in cardiomyocytes increased with age (Fig. 3c). Signature A closely resembled and was contributed dominantly by SBS5 (Fig. 3d and Extended Data Fig. 6), an age-related, ‘clock-like’ signature previously observed in many cancers^26^, normal cycling cells^6^ and single neurons^5^. This signature has been proposed to reflect faulty repair of deamination of methylated cytosines to thymine that frequently occurs in the CpG context^29^.

Signature C was distinct from the other signatures due to the prominent enrichment of C > A mutations (Fig. 3b). C > A mutations often reflect faulty repair of 8-oxoguanine (8-hydroxyguanine (8-oxo-Gua)), created by oxidative DNA damage^30^—one of the most common threats to genome stability^31^. The heart is one of the most highly metabolic organs, with large oxidative demands resulting in production of reactive oxygen species (ROS)^32^, which is thought to generate 8-oxo-Gua^33^. Decomposition of Signature C revealed significant contributions of SBS8, SBS18, SBS32 and SBS39 (Fig. 3d). SBS8 and SBS18 have been associated with the transcription-coupled repair of damaged guanine by ROS via nucleotide excision repair (NER)^34^ and base excision repair (BER)^35^ pathways, respectively, suggesting that the C > A mutations may reflect the accelerated accumulation of oxidative DNA damage that overwhelms these repair pathways.

Signature D was also enriched for C > T and T > C mutations but distinct from Signature A in its trinucleotide context (Fig. 3b). Signature D closely resembled COSMIC SBS44 (Extended Data Fig. 6) along with significant contributions from SBS6, SBS39, SBS42 and SBS46 (Fig. 3d). Both SBS6 and SBS44 have been associated with defective DNA mismatch repair (MMR) machinery and are increased in tumors associated with loss of MMR genes^26^. MMR is regulated by a small set of MMR-specific proteins in all cells^36^. Mutagenesis in the absence of one of the core MMR factors is shaped by the sequence spectrum of the unrepaired mismatches, which themselves are the product of the insertional specificity and proofreading activity of DNA polymerases. Signature D showed a striking similarity to the mutational signature of MSH6-defective human HAP1 cells, or the DLD-1 human colorectal cancer cell line, dominated by C > T and T > C mutations^37^ in a range of contexts, and small contributions of C > A, C > G, T > A, and T > G mutations. Signature D hence likely reflects a defect in the repair of damage that involves almost all mismatches.

Comparative signature analysis between cardiomyocytes, neurons, hepatocytes and lymphocytes shows shared and distinct mutational signatures (Fig. 3c). The age-dependent accumulation of Signature A was observed in all four cell types (*P* < 0.001, mixed-effects regression; Fig. 3c); we did not observe a significant difference in the rate of increase between cardiomyocytes and neurons (*P* = 0.24), which might suggest a similar ‘clock-like’ accumulation of such mutations among postmitotic cell types. Signature C also increased with age in all four cell types, but at a relatively similar rate (Fig. 3c). On the other hand, the dramatic increase in the contribution of Signature D with age was observed only in cardiomyocytes, not in neurons, hepatocytes or lymphocytes (*P* < 0.001 between cardiomyocyte and other cell types, mixed-effects regression; Fig. 3c). sSNVs that accumulated in aging cardiomyocytes showed broader substitution types with enrichment in untranscribed strands, such as T > C and T > G mutations, than those in neurons, whereas age-accumulated sSNVs in neurons showed enrichment of C > T and T > C mutations in transcribed strands (Fig. 3e). This putative MMR-related Signature D, which accumulates specifically in aged cardiomyocytes, seems to represent a distinct mutational process in the heart. The uniqueness of the signature to heart cells—both diploid and tetraploid—also argues strongly against this signature representing any sort of universal technical artifact.

### Potential sources and mechanisms of mutation formation in the aging heart.

To understand how mutations can be formed and accumulate during aging in the absence of cell cycling, it is important to recognize that both MMR, BER and NER involve steps of exonuclease removal of a segment of DNA, followed by replication of the remaining strand to reconstitute double-stranded DNA, using the nondamaged strand as template. Although oxidative base lesions are commonly repaired via the BER pathway, and NER is the main pathway for the repair of bulky adducts and other helix-distorting lesions, recent evidence has suggested a role for NER proteins in the repair of oxidative damage through interactions with BER proteins^38–40^. Our analysis of mutational signatures (Fig. 3b,d) suggested a model in which oxidative stress leads to an increased burden of damaged bases, which might overwhelm the NER, BER and MMR machinery in aged cardiomyocytes.

**Fig. 4 |:**
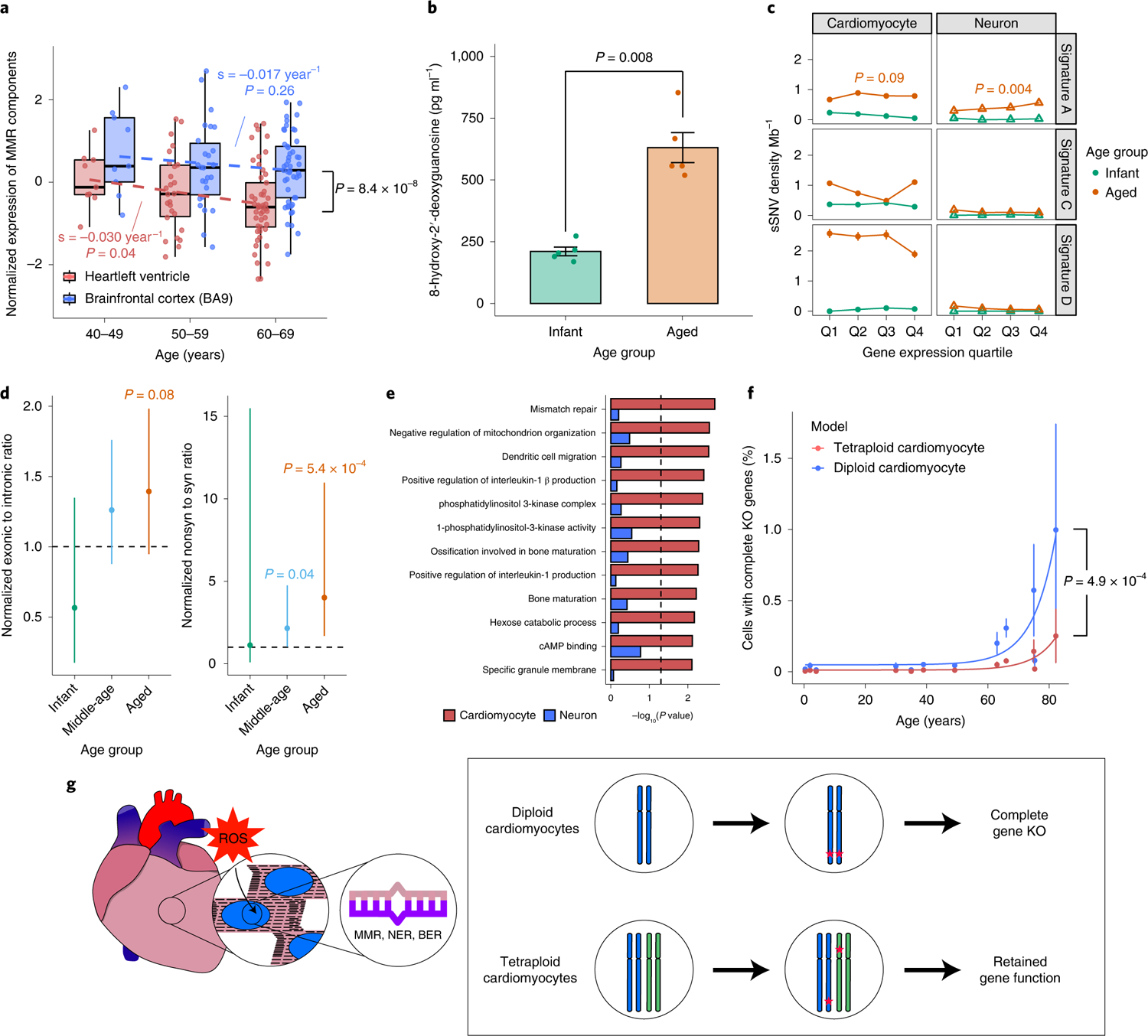
Potential mechanism of formation of sSNVs and their impact on cardiomyocytes genome during aging. **a**, mRNA expression level of MMR complex components indicating significant downregulation of MMR genes with increased age in heart samples but not in brain samples (*n* = 168 donors). Heart versus brain (*P* = 8.4 × 10^−8^, two-tailed paired Wilcoxon test); age effect in heart (*P* = 0.04, linear regression). Boxplot with whisker denotes minimum, 25%, median, 75% quartiles and maximum. **b**, Aged heart (*n* = 5 donors) showing significantly higher oxidative damage than infant heart (*n* = 5 donors), quantified by 8-OHdG assay (*P* = 0.008, two-tailed Wilcoxon test). Error bar, ± s.e.m. **c**, Association of sSNVs with gene expression level. Signature A accumulated more sSNVs in highly expressed genes in both aged cardiomyocytes and neurons (*P* = 0.09 and 0.004, linear regression). Signatures C and D are dominant in aged cardiomyocytes without significant correlation with gene expression level. *n* = 10,407 and 19,101 sSNVs of tetraploid cardiomyocytes and neurons. Error bar, ± 95% confidence interval. **d**, Aged cardiomyocytes showed excess exonic and nonsynonymous sSNVs when compared with germline mutations (two-tailed Fisher’s exact test), suggesting relaxed constraint of negative selection; *n* = 10,407 and 19,101 sSNVs of tetraploid cardiomyocytes and neurons. Error bar, ± s.e.m. **e**, Cardiomyocyte-specifically enriched GO categories (FDR-adjusted *P* < 0.05, permutation test); *x* axis denotes the enrichment *P* value for sSNVs in cardiomyocytes and neurons separately. Cardiac sSNVs are enriched in MMR pathways and pathways involved in metabolism and kinase signaling. **f**, Prediction model for the effect of sSNVs on abundance of KO tetraploid cardiomyocytes (*n* = 48 cells from 12 donors). The tetraploid model demonstrates a significantly lower KO rate than the diploid model (*P* = 4.9 × 10^−4^, two-tailed paired Wilcoxon test), especially in aged hearts, implying the protective impact of polyploidization against deleterious sSNV accumulation. Error bar, ± s.e.m. **g**, Mechanism of sSNV occurrence in heart and adaptation of cardiomyocyte to the polyploid genome. Cardiomyocytes with increased age show increased level of oxidative stress and ROS. DNA repair pathways including MMR, NER and BER in aged cardiomyocytes might not function effectively to repair this increased load of DNA damage and lead to the accumulation of sSNVs. Cardiomyocytes with higher ploidy can better tolerate the deleterious effect of these mutational burdens.

Using the RNA-seq expression data from the GTEx^41^ portal, which compiles data from 168 nondiseased donors with available heart and brain tissue gene expression profiles, we observed an overall lower gene expression for the core components of the MMR complex *(MLH3, MSH2, MSH3, MSH6, PMS1* and *PMS2*) in heart samples compared with brain samples (*P* = 2.5 × 10^−8^, two-tailed paired Wilcoxon test; Fig. 4a), and a significantly stronger decrease of those gene expression levels with aging in heart samples (*P* = 0.04, linear regression; Fig. 4a), suggesting compromised MMR activity in aged heart, except one of the MMR complex protein MLH1. Existing evidence from primary endometrial cancer studies indicates a highly variable expression pattern of MMR complex proteins even in clinical cases and the decrease of one or more MMR complex proteins is considered damaging^42^. The age-dependent decreasing of gene expression in human cardiomyocytes was further confirmed for most MMR complex genes in a recent single-cell RNA-seq dataset^43^.

**Extended Data Fig. 7 |:**
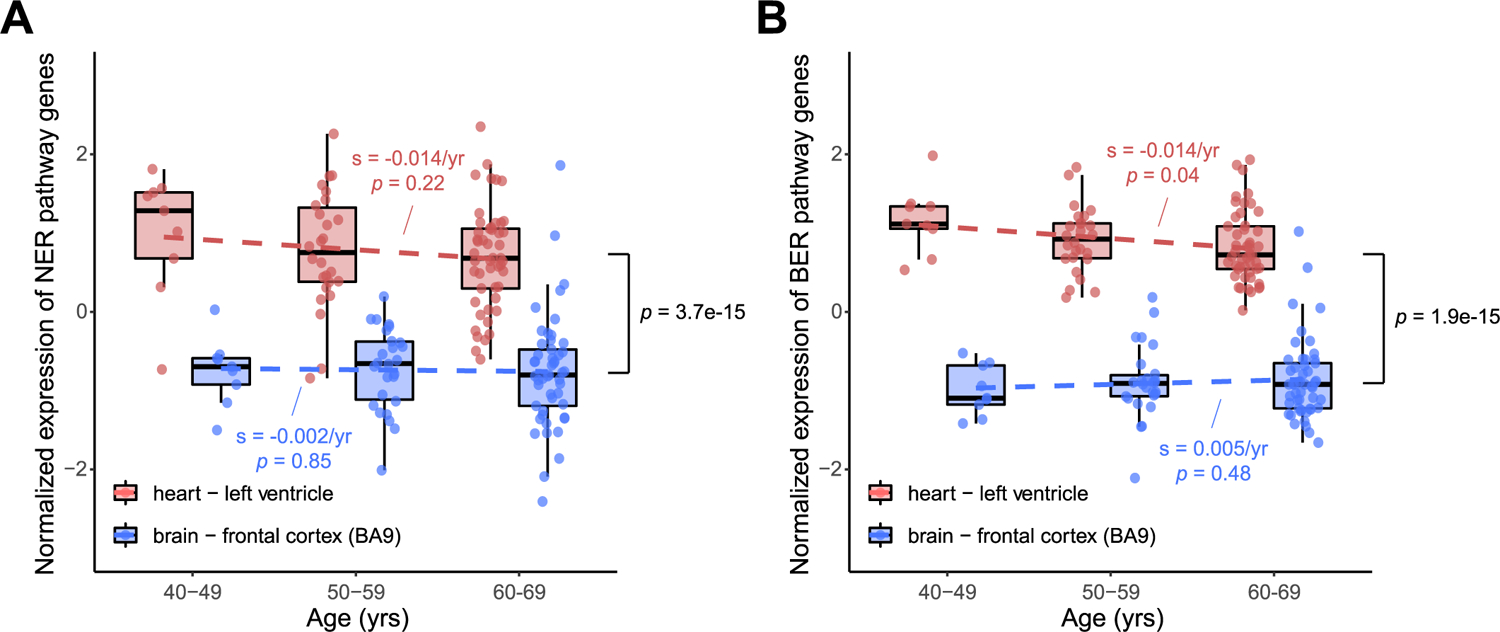
mRNA expression level of NER (A) and BER (B) pathway genes in the gTEx heart and brain samples (n = 186 donors). Heart vs. brain (*p* = 3.7 × 10^−15^ and 1.9 × 10^−15^ for NER and BER, two-tailed paired Wilcoxon test); age effect in heart (*p* = 0.04 for BER, linear regression). Boxplot with whisker denotes minimum, 25%, median, 75% quartiles, and maximum.

We also examined the expression of NER and BER pathway genes among GTEx heart and brain samples (Extended Data Fig. 7). We observed a twofold slower reduction of gene expression for NER and BER pathway genes in heart samples with age (−0.014 year^−1^; *P* = 0.22 and 0.04) than the MMR pathway (−0.030 year^−1^). This suggested that the MMR pathway might be affected more severely during the aging of cardiomyocytes, which could potentially explain the cardiomyocyte-specific accumulation of sSNVs from the MMR-related Signature D.

Under physiological conditions, ROS are scavenged by the antioxidant system, but when the ROS concentration is very high, oxidative damage occurs to DNA. Guanosine is the most oxidized among the DNA nucleobases, with 8-hydroxy-2-deoxyguanosine (8-OHdG) being a product of oxidative DNA damage and considered as a biomarker of oxidative stress. High levels of 8-OHdG have been correlated with various age-related cardiovascular disease progression^44^, but the exact causal relationship has not been fully elucidated. We directly assessed potential oxidative damage in the left ventricular cardiomyocytes (*n* = 10, five infant and five aged donors) using an enzyme-linked immunosorbent assay (Methods). We found that the level of 8-OHdG in aged hearts was more than twice as high as that in infant hearts (*P* = 0.008, two-tailed Wilcoxon test; Fig. 4b). These data suggest that increased oxidative stress leads to elevated 8-OHdG, which may overwhelm DNA repair systems, and contributes, at least in part, to the increased DNA mutational burden.

### Functional impact of sSNVs in the aging process.

**Extended Data Fig. 8 |:**
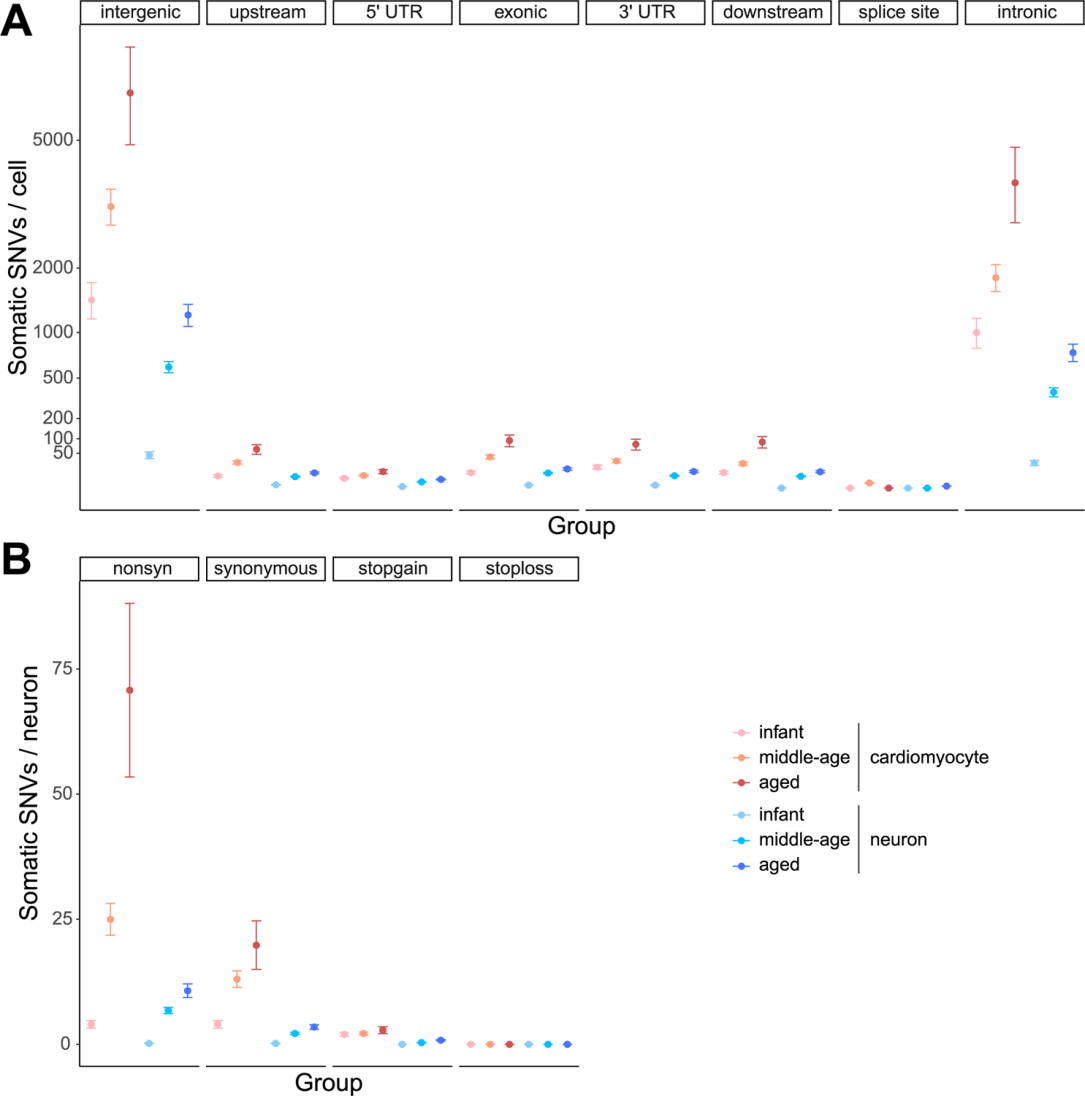
Distribution of sSNVs across different categories of genic annotation **(A) and mutation type (B).** sSNVs identified from tetraploid cardiomyocytes (n = 10,407) and neurons (n = 19,101) were grouped according to the age group of donors. Error bar, mean ± 95%CI.

To further investigate whether the occurrence of sSNVs is associated with defective gene transcription and function, we stratified and compared cardiac and neuronal sSNVs by using the expression profiles of corresponding tissues in GTEx. By estimating the mutational signature composition of genic sSNVs in each gene expression quartile and for each age group, we found that Signature A sSNVs are enriched in highly expressed genes at a similar level in both aged cardiomyocytes and aged neurons (Fig. 4c, upper), suggesting Signature A as a common transcription-associated signature^45^. In contrast, Signatures C and D showed higher contributions in aged cardiomyocytes than in aged neurons, without a strong association with gene transcription level (Fig. 4c, middle and lower), indicating that Signatures C and D might result from mechanisms distinct from Signature A, and relatively specific to cardiomyocytes. We found a higher proportion of exonic and nonsynonymous mutations in aged cardiomyocytes when compared with germline heterozygous SNVs (Fig. 4d and Extended Data Fig. 8), which could be explained by the relaxed constraint of negative selection in the somatic context. Gene ontology (GO) analysis found that genes involved in mismatch repair, mitochondria organization and phosphatidylinositol 3-kinases (PI3Kinase) pathways showed sSNV enrichment in aged cardiomyocytes (false discovery rate (FDR)-adjusted *P* < 0.05, permutation test; Fig. 4e). More specifically, we observed protein-altering deleterious somatic mutations in kinase pathway genes of aged cardiomyocytes (Supplementary Table 5), such as *WNK2* (ref. ^46^), *TAOK3* (ref. ^47^) and *BAZ1B*^48^, which play key roles in the regulation of electrolyte homeostasis, cell signaling survival, proliferation activities, and DNA damage response. Additionally, protein-altering mutations were identified in *ZMYM6* (ref. ^49^) and *DOCK6* (ref. ^50^) that are associated with cytoskeletal organization (Supplementary Table 5).

Although many heterozygous mutations would likely compromise cardiomyocyte function, it is expected that deleterious gene ‘knockout’ (KO) mutations in haploinsufficient genes would be especially damaging if all the alleles are affected, and that there may be a threshold for the accumulation of such KOs above which cardiomyocyte function would deteriorate. Higher ploidy cells with more copies of chromosomes may potentially represent adaptive mechanisms to guard cells against these KO mutations. Therefore, we compared the accumulation of gene KOs in diploid versus tetraploid cardiomyocytes using a prediction model. In this model, at least two coincident deleterious sSNV events in a diploid cell, or four deleterious sSNVs within one gene in a tetraploid cell, cause loss of function. Diploid cardiomyocytes had an average probability of 0.2% of getting one or more genes completely knocked out by age 60 years, with this probability increasing to 1% by age 80 years, implying that a substantial fraction of cardiomyocytes would carry damaging mutations (Fig. 4f). In contrast, tetraploid cardiomyocytes showed a significantly lower probability of gene KO (*P* = 4.9 × 10^−4^, two-tailed paired Wilcoxon test), with less than 0.2% of cells with KO genes at age of 80 years (Fig. 4f). These data strongly suggest that tetraploid cardiomyocytes are more effective in averting the loss of gene function caused by age-related mutations (Fig. 4g).

## Discussion

Our data show that each individual cardiomyocyte has a profoundly distinctive genome, harboring sSNVs accumulated throughout the lifetime. After controlling for the genomic size difference between tetraploid and diploid cells, cardiomyocytes accumulate age-related SNVs at rates higher than neurons and lymphocytes, but similar to hepatocytes. This finding indicates that the heart and liver, two highly metabolic active organs, harbor a higher load of somatic mutations and have the tenacity to become polyploid, potentially to endure oxidative stress. Although the accumulation rates of hepatocyte and cardiomyocyte are similar, cardiomyocyte sSNVs display distinct signatures of mutagenic processes, with a predominant contribution of Signature D that has been associated with defects in the MMR pathway.

Existing literature shows that cardiomyocyte genome ploidies and nuclear counts vary widely across different mammalian species^19^. Polyploidization is a characteristic feature of mammalian cardiomyocytes and can be stress-induced and/or developmentally programmed^51,52^. Polyploidization not only plays a role in increasing cell size and metabolic output, but also promotes alterations in the transcriptome and metabolome. Polyploidy also frequently confers resistance to environmental stresses not tolerated by diploid cells. Our prediction models show that tetraploid cardiomyocytes have a significantly lower probability of complete gene KO compared with diploid cardiomyocytes, indicating that cardiomyocyte polyploidization potentially offers a mechanism to ameliorate the deleterious effects of this rapid mutation accumulation. Human cardiomyocytes initially are mainly diploid, though tetraploid cardiomyocytes begin to appear soon after birth. The formation of hexaploid, octoploid and potentially higher ploidy cells arises with advanced age.

Our data indicate that aging results in increased generation, decreased repair, or both, of oxidative DNA lesions. Age-related myocardial sSNVs have distinctive C > A mutations, which are hallmarks of oxidative damage, and direct quantification by enzyme-linked immunosorbent assay shows increased oxoguanine in the aged heart. Decomposition of Signature C with COSMIC signature indicates significant contributions of SBS8 and SBS18. This finding strongly suggests that the damaged guanine accumulates either via disrupted NER, linked to SBS8 as well as disrupted BER, linked to SBS18. Most likely these C > A mutations are from the accelerated accumulation of oxidized nucleotides that overwhelm these repair pathways, since our finding from GTEx expression data indicates expression of both NER and BER pathway genes are changed only mildly during heart aging. Oxidized guanine nucleotides reflect the presence of increased ROS, which has previously been associated with various cardiovascular diseases, and can be generated by a variety of processes including inflammation and mitochondrial dysfunction that are well studied in cardiovascular disease. Our findings suggest that increased oxidative stress leads to elevated 8-OHdG, which may overwhelm NER and BER repair machinery, resulting in increased DNA mutational burden. Another mutational signature that is uniquely enriched in cardiomyocytes compared with neurons suggests a role of defective MMR in mutation generation. It is known that failure of DNA MMR is associated with a strikingly elevated rate of base substitution mutations and, as a consequence, tumors with MMR deficiency are amongst those with the highest number of somatic mutations. Our findings of increased sSNV counts in the aged heart suggest that MMR may not be efficient at correcting mismatched nucleotides in aged cardiomyocytes, contributing to the large increase in sSNVs.

Here we show that the number of sSNVs and the likelihood of disrupting essential gene function in human cardiomyocytes increases significantly with age, suggesting that the age-related cellular dysfunction in aged cardiomyocytes could be due partially to somatic mutations, although more studies will be needed to draw a causal relationship between mutational burden and age-associated decrease in cardiac function. We identified a total of 10,407 sSNVs from 48 tetraploid cardiac nuclei by LiRA. Among 75 coding sSNVs, 36 were predicted to be damaging (Supplementary Table 5). We also observed seven sSNVs shared by two cardiomyocytes (no sSNVs shared by three or more cardiomyocytes); six of these were shared by cardiomyocytes obtained from the same donor, indicating early somatic mutations in the common lineage of these cardiomyocytes. More comprehensive measurements of somatic mutation from healthy and diseased myocardium will be needed to decipher the functional impact of somatic mutation in aging and heart disease. In this study, we investigated only single nucleotide variants, but cardiomyocytes could also carry other types of somatic mutations, including indels and structural variations. MDA requires extensive quality control to identify well-amplified samples, so that our samples are biased towards cells that amplified well and evenly, that is, cells more likely to have intact genomes. Importantly, the sSNV increase rate in neurons amplified by MDA (19 sSNV per cell genome per year) is consistent with the rate estimated by a recently developed duplex sequencing protocol^6^ without genome amplification (20 sSNV per cell genome per year), suggesting the accuracy of MDA-based sSNV analysis. Newer methods, such as primary template amplification (PTA)^7^ or META-CS^53^ may provide an improved means to study somatic mutations in diploid and multiploid cardiomyocytes with broader genomic coverage for variant calling and better distinction of sSNVs from single-stranded lesions. Nevertheless, these results provide an early view into the mutational landscape of terminally differentiated cardiomyocytes. Our analysis of human cardiomyocytes lays a foundation for better understanding of the genomic landscape and mechanisms driving mutation accumulation in aging cardiomyocytes that may help develop new treatments to reduce age-related cardiomyocyte dysfunction.

## Methods

### Human tissues and DNA sample preparation.

This study was approved by the Boston Children’s Hospital institutional review board. Samples were processed according to a standardized protocol under the supervision of the National Institutes of Health (NIH) NeuroBioBank ethics guidelines. Research on these deidentified specimens and data was performed at Boston Children’s Hospital with approval from the Committee on Clinical Investigation (S07-02-0087 with waiver of authorization, exempt category 4). All human tissues were obtained from the NIH NeuroBioBank at the University of Maryland. Once we received the tissue from the BioBank, DNA degradation evaluation was performed by isolating DNA from the tissue and performing gel electrophoresis as well as a Genomic DNA Screen Tape Station. Tissues with fragmented DNA were not selected for further studies.

### Statistics and reproducibility.

No statistical methods were used to predetermine sample size. The experiments were not randomized, and the investigators were not blinded to allocation during experiments and outcome assessment.

### Isolation of cardiomyocyte nuclei.

Cardiac nuclei were isolated using a density sedimentation protocol^13^. Briefly, 100 mg of cardiac tissue from the left ventricle was homogenized in 5 ml of ice-cold lysis buffer (0.32 M sucrose, 5 mM CaCl_2_, 3 mM magnesium acetate, 2.0 mM EDTA, 0.5 mM EGTA, 10 mM Tris-HCl (pH 8.0) and 1 mM dithiothreitol (DTT)). The suspension was further dounced (20 strokes) with a type A pestle in a glass douncer (Sigma). The homogenized solution was filtered through 100 and 70 μm strainer (Pluriselect) and centrifuged for 7 min at 700*g* at 4 °C and the crude nuclear pellets were resuspended in 2.1 M sucrose solution (2.1 M sucrose, 3 mM magnesium acetate, 1 mM DTT and 10 mM Tris-HCl, pH 8.0). This was then layered onto a cushion of 5 ml 2.1 M sucrose solution and centrifuged at 30,000*g* for 1 h at 4 °C. The pellet from each tube was then resuspended with 1.5 ml nuclei storage buffer (0.43 M sucrose, 70 mM KCl, 2 mM MgCl_2_, 10 mM Tris-HCl (pH 7.2) and 5 mM EGTA) for further analysis.

### Flow cytometry.

Accurate identification of cardiomyocyte nuclei is crucial for the analysis of this study. Single cardiac nuclei were isolated using FANS-based cardiac troponin T staining and nuclear DAPI intensity, using FACSAria (20 psi, 100-mm nozzle, Becton Dickenson Biosciences). Cardiac nuclei were identified using a sequential gating strategy. Initial size gates for FSC versus SSC were set to select the large cardiac nuclei with high FSC and SSC corresponding to larger and more granular cells. Cell doublet discrimination was performed by a combination of high forward scatter height and area FSC-H/FSC-A and SSC-H versus SSC-W plots. H versus W or A allows separating the doublets from the single-cells containing 4n amounts of DNA. Cells containing 4n amounts of DNA have double the A and H values whereas W is roughly the same as cells containing 2n amounts of DNA. Cardiomyocytes are the only tetraploid cell in cardiac tissue, so ploidy is a convenient way to purify them. However, to rule out any possibility that tetraploid cells accumulate mutations in different ways from diploid cells, or that the amplification, sequencing and calling process performs differently in tetraploid cells, we carried out replicate analysis of diploid cells by isolating diploid cardiomyocytes using cardiomyocyte-specific markers (cardiac troponin T). The genomes of every single nucleus were amplified using MDA^20^.

### Ploidy quantification by imaging flow cytometry and karyotyping.

We used a FlowSight Imaging Flow Cytometer to combine the advantages of traditional flow cytometry and microscopy to verify the cardiac nuclei ploidy. Left ventricular cardiomyocyte nuclei were isolated and stained with Hoechst (Invitrogen, catalog no. H3570; 1:1,000) and the cardiomyocyte marker cardiac troponin T (Abcam, catalog no. ab56357; 1:250). The nuclei were resuspended in PBS at a concentration of 2 × 10^7^ cells ml^−1^. The DNA content was detected by a FlowSight Imaging Flow Cytometer (Luminex), equipped with a ×20 objective lens, and analyzed by image analysis software (IDEAS). The percentage of 2n, 4n and greater than 4n ploidy was determined by setting gates using the calibration with nuclei of noncardiomyocytes at 2n. The software separated the cardiomyocyte nuclei using brightfield, Hoechst and Troponin T images.

Chromosomes were visualized by Giemsa staining (GIBCO KaryoMAX Giemsa Stain Stock Solution, catalog no. 10092–013) according to the manufacturer’s protocol. Briefly, sorted cardiac nuclei from 1465 were treated with a hypotonic solution (0.075 M KCl) and preserved in their swollen state with Carnoy’s fixative; further nuclei were dropped on to slides and air-dried. The slides were stained with freshly prepared Giemsa staining solution (3:1 ratio of Gurr Buffer and Giemsa Stain) and visualized at ×100 magnification.

### Expression analysis of sorted cardiac nuclei.

ddPCR assays were performed to test the cardiomyocyte-specific gene expression by using Tagman probe of *PLN* (Hs01848144), *TNNT2* (Hs00943911), *MYH7* (Hs01110632), *CD31* (Hs00169777_m1) and *PDGFB* (Hs01019589). QX100 Droplet Digital PCR System (Bio-Rad) was used with standard parameters. We measured numbers of droplets that were positive and negative for each gene using QuantaSoft software.

### Library preparation and WGS.

**Extended Data Fig. 9 |:**
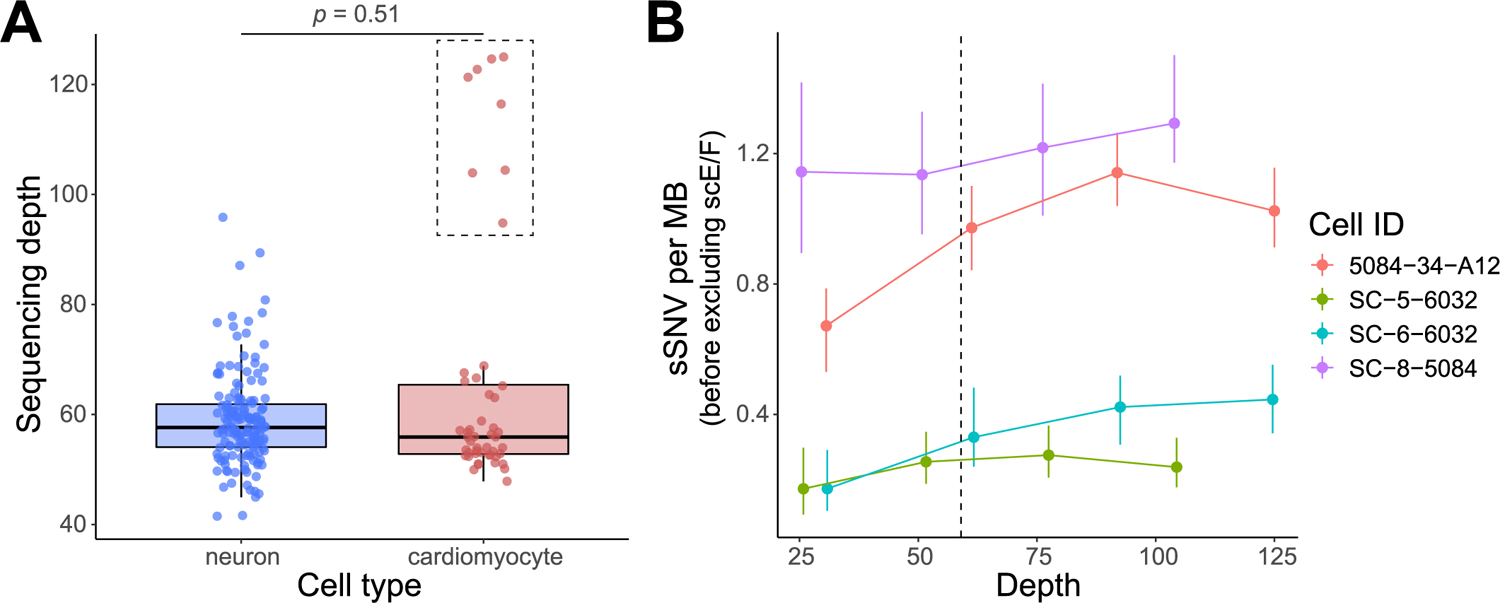
Sequencing depth and down-sampling performance for cardiomyocytes and neurons. **A**, No systematic difference in sequencing depth (*p* = 0.51, two-tailed Wilcoxon test) between tetraploid cardiomyocytes (n = 48) and neurons (n = 155). Eight outlier cardiomyocytes (in the dashed rectangle) were intentionally sequenced at doubled sequencing depth from two donors. Boxplot with whisker denotes minimum, 25%, median, 75% quartiles, and maximum. **B**, LiRA-estimated sSNV burden remained generally robust with varied sequencing depths at or above the average depth in neurons, denoted by the dashed line. Four outlier cardiomyocytes from (**A**) were randomly chosen, and their sequencing reads were *in silico* down-sampled into 25%, 50%, and 75% of the original sequencing depths. Error bar, mean ± 95%CI.

We sequenced 48 tetraploid and 8 diploid single cardiac nuclei from 12 individuals with ages from 0.4 to 82 years, including three infant (<4 years), six middle-aged (30–66 years) and three aged (>75 years) individuals (Table 1). From each of the 12 individuals, we sequenced four single cardiac nuclei. Low coverage library preparations were carried out according to the manufacturer’s instructions (QIAseq FX single-cell DNA Kit). MDA-amplified DNA libraries were prepared with the Illumina TruSeq Nano LT sample preparation kit. Bulk DNA was extracted using the QIAamp DNA Mini kit with RNase A treatment. Paired-end sequencing (150 bp × 2) was performed on a HiSeq ×10 instrument. Single-cell and bulk WGS library preparations and sequencing were done at Macrogen Genomics. The sequencing depth of cardiomyocytes was comparable with that of neurons we previously studied (Extended Data Fig. 9).

### Read alignment and postprocessing.

Reads generated from single-cell WGS were aligned against the GRCh37 human reference genome by BWA (v.0.7.15) with default parameters. Duplicate reads were masked by Mark Duplicate of Picard (v.2.8) and then postprocessed with local realignment around indels and base quality score recalibration using Genome Analysis Toolkit (GATK) (v.3.5).

### Measuring the evenness of genome amplification in single-cells.

We measured the evenness of genome amplification in single-cells using two metrics: MAPD and CoV. MAPD is the median value of absolute differences between the copy number ratios of neighboring bins with variable lengths, where bins were divided to have the same number of uniquely mapping reads. CoV is the measure of variance of bin-wise copy number ratios, calculated by taking the ratio of their standard deviation to the mean. Both higher MAPD and CoV scores represent greater unevenness of single-cell genome amplification. Binning, GC normalization, segmentation and copy estimation were performed following the previous single-cell copy number analysis protocol^54^, to obtain the copy number ratio per bin and calculate MAPD and CoV scores.

### Estimation of amplification dropout rates in single-cell WGS data.

Germline heterozygous SNVs were identified from bulk WGS data using HaplotypeCaller of GATK with default parameters (-stand_call_conf 30.0 -stand_emit_conf 30.0 -ploidy 2)^55^, and only those reported by the 1000 Genomes Project were considered subsequently as high-confidence variants to estimate dropout rate. For each single-cell, a germline heterozygous SNV was considered as locus dropout if the total coverage in single-cell WGS is less than five times and considered as allelic dropout if the number of reads supporting either a reference or a mutant allele is less than two. The genome-wide locus- and allele-dropout rates were then calculated as the proportions of dropout sites among all germline heterozygous SNVs.

### Somatic SNV calling from single-cell WGS data.

We performed phasing-based linked read analysis using the LiRA method (v.2018Feb)^21^ to identify sSNVs in single-cells using around 30× WGS data of nonheart tissue from the same individual as bulk germline controls. Served as the input of LiRA, SNVs from each single-cell and bulk sample were called using the HaplotypeCaller from GATK with default parameters (-stand_call_conf 30.0 -stand_emit_conf 30.0 -ploidy 2); germline SNVs identified from bulk samples were further phased by Shapeit 2 (v.904)^56^. LiRA distinguishes true somatic mutations from base-calling or amplification errors by leveraging the linkage information between the somatic candidate and adjacent phased germline mutations. For each single-cell, the threshold for number of phasable reads supporting mutant allele was calculated to control for more than 90% true positive rate. The detection sensitivity in phasable regions was estimated from germline SNVs and then used to adjust the LiRA-detected sSNV count to the real count. sSNV density per megabase was calculated for each single-cell with the consideration of doubled genomic size for tetraploid cardiomyocytes. We considered sSNVs in autosomes only to avoid potential detection bias in sex chromosomes between different genders.

### Somatic SNV burden correction for tetraploid cells.

**Extended Data Fig. 10 |:**
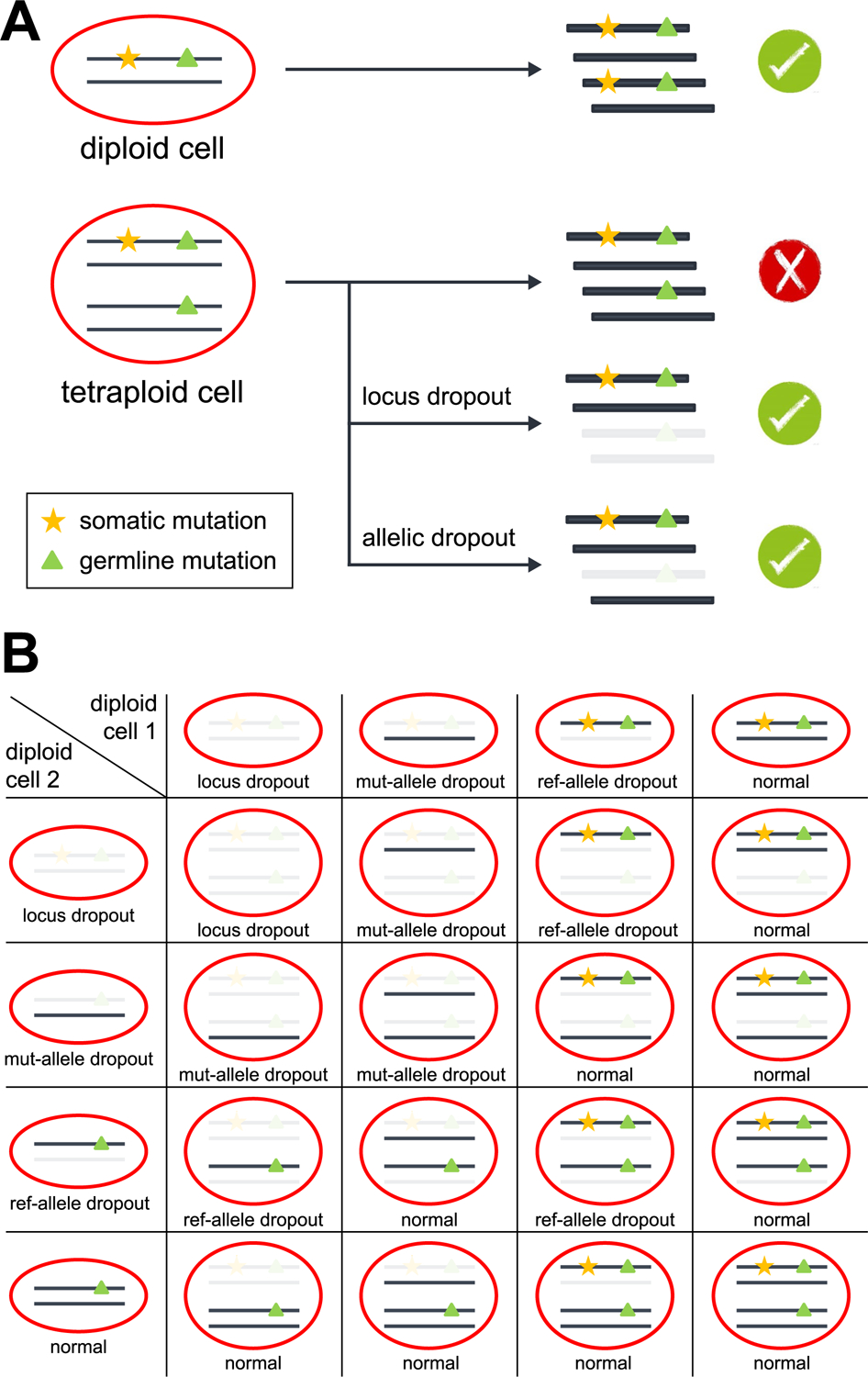
Model for calling sSNVs from diploid and tetraploid cells by LiRA. **A**, In diploid cells, LiRA identified the complete linkage between each sSNV candidate and its adjacent germline heterozygous mutation, which distinguishes true sSNVs from technical artifacts. In tetraploid cardiomyocytes, sSNVs present in one out of the four haplotypes were able to be called by LiRA when the reads violating complete linkage were lost due to allelic or locus dropout. **B**, Dropout status of a tetraploid cell determined by its two diploid origin cells. Rows and columns denote the dropout status of two diploid origin cells, respectively. mut-allele, mutant allele of the germline mutation; ref-allele, reference allele of the germline mutation.

Each tetraploid cardiomyocyte contains two sets of diploid genomes (that is, four haplotypes). Theoretically, somatic mutations present in one out of the four haplotypes can be called by LiRA only when the reads violating complete linkage were lost due to allelic or locus dropout (Extended Data Fig. 10a). In comparison, germline mutations can be called in the same way as in the diploid cells. Therefore, the LiRA-estimated power from germline SNVs should be corrected by cell-specific allelic or locus dropout rates before applying to the count of identified sSNVs in tetraploid cells.

Theoretically, the dropout status of a site in a tetraploid cell (*S*_4n_) was determined by the status of its two diploid origin cells (Extended Data Fig. 10b): a locus dropout happened in a tetraploid cell only when both of its diploid origin cells were locus dropout, whereas an allelic (reference or mutant allele) dropout happened in a tetraploid cell when two copies of the corresponding alleles from two diploid origin cells were lost by locus or allelic dropout. Using simulated tetraploid cells by an in silico mixture of single-cell WGS data from two diploid cells from the same individual, we observed that the locus dropout rate in simulated tetraploid cells generally equals the product of the dropout rates in the two original diploid cells, suggesting independence of dropout occurrences across the genome between MDA-amplified cells.

Cell-specific allelic and locus dropout rates were estimated for each tetraploid cell, and the dropout rate for its two diploid origins was then calculated from the above matrix under the assumption of equal rates between the two diploid origins. The count and density of LiRA-called sSNVs were further adjusted by the locus and allelic dropout rate of the corresponding diploid origins and the sensitivity loss due to the decrease of per-haplotype sequencing depths from diploid to tetraploid cells.

### Mutational signature analysis.

Mutational signatures were decomposed de novo by the NMF-based mutational signature framework^27^ using MutationalPatterns (v.1.8.0), using the 96 trinucleotide contexts of sSNVs detected from tetraploid cardiomyocytes in this study as well as nondisease neurons that were previously studied^10^. For most of the downstream analyses, except the regression modeling of sSNV density where we used all neurons to cover the whole age span, only neurons from the same donors of the studied cardiomyocytes were considered to better control for donor background. We estimated signature stability and reconstruction error and found that four signatures best fit the observed sSNV profiles (Extended Data Fig. 4). We then compared our de novo signatures (Signatures N1, N2, N3 and N4) with previously reported signatures in neurons (Signatures A, B and C)^10^ and signatures potentially resulting from MDA artifacts (SBS, scE and scF)^22^. As shown in Extended Data Fig. 5, Signatures N1, N2 and N3 resemble Signature B (as well as SBS scF), A and C, respectively, whereas Signature N4 did not show high similarity to any of these signatures, suggesting a potential cardiac-specific signature (renamed as Signature D).

To remove the potential contamination of MDA artifacts, we decomposed the sSNV profile of each single-cell into Signatures N2/A, N3/C, N4/D as well as SBS scE and scF (Signature N1/B was not included because it was nearly identical to SBS scF) using MutationalPatterns. We then calculated the signature-specific sSNVs density for each single-cell by multiplying the overall sSNV density and the cell-specific weight for each signature. The contributions of SBS scE and scF were subtracted from the overall sSNV density for subsequent burden analyses.

### Burden and list of sSNVs in hepatocytes and lymphocytes.

Genome-wide sSNV burden and list of MDA-amplified hepatocytes and lymphocytes profiled by single-cell WGS were extracted from Brazhnik et al.^24^ and Zhang et al.^25^, respectively. The contributions of SBS, scE and scF were estimated and subtracted from the overall sSNV burden for each hepatocyte and lymphocyte, following the same strategy as that performed in each cardiomyocyte and neuron.

### Mixed-effects modeling of somatic SNV density.

To study the age-dependent somatic mutation accumulation and the rate difference between cardiomyocyte and other cell types, we performed linear mixed-effects regression modeling using the lme4 (v.1.1–23) and lmerTest (v.3.1–2) R packages. Overall and signature-specific sSNV density per megabase was modeled as continuous outcomes. Age was modeled as a fixed effect, whereas donor and cell type were modeled as random effects, because cells from the same donor and cell type may share the biological environment and thus be independent in terms of sSNV density. The maximum likelihood method was used to fit linear mixed-effects regression models.

To test the age effect of sSNV density in healthy individuals, we fitted the model *y*_*ij*_ = *β* × *ρ*_*j*_ + *μ* + *θ*_*j*_ + *ε*_*ij*_ where *y*_*ij*_ is the sSNV density in cell *i* of donor *j*, *β* is the fixed effect of age, *ρ*_*j*_ is the age of donor *j*, *μ* is the number of sSNVs at birth, *θ*_*j*_ is the random effect of each donor following a normal distribution with mean 0 and variance *τ*, and *ε*_*ij*_ is the measurement error of each cell following a normal distribution with mean 0 and variance *σ*_*ij*_. We observed a significant age-association of sSNV density in tetraploid cardiomyocytes (*P* = 7.3 × 10^−4^; Fig. 2b), and further confirmed similar sSNV densities between tetraploid and diploid cardiomyocytes (Extended Data Fig. 2).

To test the difference in the age effect between different cell types, we fit the model *y*_*ijk*_ = (*β* + *γ*_k_) × *ρ*_*j*_ + *μ* + *θ*_*jk*_ + *ε*_*ijk*_ where *y*_*ikj*_ is the sSNV density in cell *i* from cell type *k* of donor *j*, *β* is the fixed effect of age, *γ*_*k*_ is the fixed effect of cell type *k* on age (interaction terms between age and cell type), *ρ*_*j*_ is the age of donor *j*, *μ* is the number of sSNVs at birth, *θ*_*jk*_ is the random effect of the donor-cell type pair following a normal distribution with mean 0 and variance *τ*, and *ε*_*ijk*_ is the measurement error of each cell following a normal distribution with mean 0 and variance *σ*_*ijk*_. As shown in Fig. 2e, we observed that cardiomyocytes showed an age effect that was significantly larger than neurons and lymphocytes (*P* = 5.0 × 10^−3^ and 8.7 × 10^−4^) but not hepatocytes (*P* = 0.53).

To control for potential confounding factors in sSNV detection, we introduced *δ*_*ij*_ into the previous models, where *δ*_*ij*_ denotes one of the potential confounding factors including MAPD and CoV scores (measurement of amplification evenness), sequencing depth, library insert size, number of heterozygous germline SNVs and PMI of donors. We confirmed that the age-dependent increase in cardiomyocytes and the cardiomyocyte-neuron difference remained statistically significant (*P* < 0.05), suggesting that the sSNV accumulation pattern we found in cardiomyocytes was very unlikely to be caused by these technical issues.

### Mutation spectrum and strand bias analysis.

The LiRA-identified sSNVs were grouped into three categories according to the age of their cell donor: infant (<4 years), middle-aged (30–66 years) and aged (>75 years), and then the mutation spectrum and strand bias were calculated for each age category. The transcriptional strands of genic sSNVs were assigned based on the UCSC TxDb annotations by MutationalPatterns^57^, where mutated bases (‘C’ or ‘T’) on the same strand as the gene direction were categorized as ‘untranscribed’ and on the opposite strand as ‘transcribed.’ To characterize sSNV accumulation during aging, we further estimated the mutation spectrum and strand bias for the net increase of sSNVs between infant and aged categories. Specifically, we first measured the absolute sSNV count for each mutation type by multiplying its proportion and the average sSNV burden for each age category, and then subtracted the sSNV count for each mutation type between infant and aged categories. The statistical significance of strand bias was determined by the Poisson test.

### Gene expression analysis.

The expression matrix ‘Gene read counts’ (GTEx Analysis v.8) for left ventricle of the heart and frontal cortex (BA9) of the brain was downloaded from the GTEx portal, since these two regions are the corresponding source tissues for our single-cell cardiomyocytes and neurons, respectively. The per-gene expression value was then normalized for each tissue sample after estimating sample-specific size factor and dispersion as well as modeling tissue, age and gender factors using DESeq2 (ref. ^58^) (v.1.24.0) with the recommended protocol and default parameters. To study the age-dependent changes in MMR activity in the heart and brain, we extracted the expression levels of core components of the MMR complex (*MLH1*, *MLH3*, *MSH2*, *MSH3*, *MSH6*, *PMS1* and *PMS2*) as well as genes in the NER and BER pathways annotated by the KEGG database^59^. Individuals with both heart and brain expression profiles were binned according to their ages. Individuals with ages less than 40 years and more than 70 years were excluded due to the small sample size (*n* ≤ 10). For the remaining 168 individuals, we calculated the average expression levels of the MMR, NER and BER genes in heart and brain samples, separately, and tested their association with age using the linear regression model.

To investigate the relationship between somatic mutation and gene expression, we assigned genes into four quartiles based on their average expression values in heart or brain samples across all GTEx individuals. Cardiac and neuronal sSNV densities were calculated for each quartile of genes, after normalizing by gene length and per-cell sSNV detection power. The standard deviation of sSNV density was estimated using a permutation test, in which the quartile classification of genes was shuffled randomly and the permuted sSNV densities were calculated for 1,000 rounds. We further performed an NMF-based mutational signature decomposition for sSNVs located in each quartile of genes, to estimate the relative contributions of Signature A, Signature C, Signature D, SBS scE and SBS scF for each quartile. The sSNV density for each signature was calculated by multiplying the overall sSNV density by the signature contribution. We also performed the above analysis by using the expression profiles from aged individuals (>75 years) only and observed robust results.

### Functional enrichment analysis.

GO functional enrichment analysis was performed using GOseq (v.1.34.1). We assigned a binary value ‘0’ or ‘1’ to each RefSeq gene according to whether any sSNV was present in the gene in any single-cell and built the sSNV-gene table for cardiac and neuronal sSNVs separately. GOseq uses the Wallenius approximation method to test the enrichment of sSNVs for each GO term, after applying a probability weighting function to control for potential bias from gene length. Genes without any GO annotation were ignored when calculating the total gene count. GO terms with fewer than five member genes with sSNVs were excluded to avoid ascertainment bias. GO terms with more than 1,000 member genes were also excluded.

To identify GO terms that were specifically enriched in cardiomyocytes but not in neurons, we performed a permutation test among all GO terms with *P* < 0.01 for either cardiac or neuronal sSNVs. For each permutated GO term, we compared the observed rank difference in GOseq’s *P* between cardiac and neuronal sSNVs against the expected null distribution, which was estimated by 1,000 rounds of random shuffling of the sSNV-gene tables. The FDR method was applied for correcting multiple hypothesis testing.

### Measurement of oxidative stress.

The level of 8-OHdG and 8-oxo-Gua was measured in 250 ng total nucleic acids extracted from left ventricular cardiomyocytes using a competitive enzyme-linked immunosorbent assay kit (Cayman Chemical, catalog no. 589320) according to the manufacturer’s instructions. The samples were incubated for 1 h with a monoclonal antibody against 8-OHdG in a microtiter plate precoated with 8-OHdG and 8-oxo-Gua. The final color was developed by the addition of 3,3,5,5-tetramethylbenzidine, and absorbance was measured at 450 nm. The samples were diluted at 1:50 with enzyme immunoassay buffer before assaying.

### Modeling accumulation of gene KOs in cardiomyocytes.

Accumulation of exonic, deleterious gene KO mutations might be detrimental to proper cell function. These mutations can be ‘biallelic’ in the case of diploid cells, or ‘quadallelic’ in the case of tetraploid cells. The number of sSNVs identified in this study were used to estimate the accumulation of gene KOs in single-cells, using an extension of the method described in Lodato et al.^5^. To account for genes that are highly dosage sensitive, and thus can be haploinsufficient, we included a factor to capture the probability of a mutation landing on an allele of a gene with a high pLI score. The pLI metric measures the probability of loss-of-function intolerance^60^, and genes with pLI score greater than 0.90 are considered highly dosage sensitive. These high pLI score genes comprise 17% of all genes. Consequently, the calculation was computed as follows:

$$n=\text{ number of estimated sSNVs }\times \frac{\text{ total number of deleterious variants }}{\text{ total number of variants }}\times p$$

$$d_{i}=\left\{\text{ event that gene i has at least one mutation }\right\}$$

$$\pi_{i}=\left\{\text{ event that gene i has a high pLI score }\right\}$$

D={ probability of a gene having a deleteriousmutation }

$$\text{Pr}(\text{KO}|\pi ,D,n)=\pi \times \left(1-{\left(1-D\right)}^{n}\right)+(1-\pi )\left(1-{\text{e}}^{-nD}\right)$$

where *p* is the ploidy factor that captures the probability of obtaining a mutation on the remaining alleles of the gene (for example, *P* = 0.5 for diploid genomes, and *P* = 0.125 for tetraploid genomes) and *n* is the expected number of deleterious mutations. Considering the similar sSNV density but halved genomic size in diploid cardiomyocytes when compared with tetraploid cardiomyocytes (Extended Data Fig. 2), the genome-wide sSNV burden in diploid cardiomyocytes of each individual was calculated as 50% of the burden in corresponding tetraploid cardiomyocytes. The mean and s.e.m. were calculated across all cells per individual, for tetraploid and diploid cardiomyocytes separately. Regressions were performed using an exponential model to capture the nonlinear trend of the probability of obtaining cells with KO genes with age. All calculations were performed using custom R scripts.

### Reporting summary.

Further information on research design is available in the Nature Research Reporting Summary linked to this article.

## Extended Data

## Supplementary Material

Table_S1_Ploidy Case Info

Table -S5_heart exonic mutation

table _S4_Mutational rate info

Table _S2_Sequencing info

Table _S3_Mutational Site info

## Figures and Tables

**Table 1 |:** Case information analyzed in this study for sequencing

Case ID	Age (years)	Height (inches, range)	HW (g)	BW (lb, range)	Sex	Diagnosis	Cause of death	Hypertrophy/fibrosis?	RIN	PMI (h)
1278	0.4	20–25	42	15–20	M	Normal	SIDS	No	NA	8
1864	2	35–40	59	30–35	F	Normal	Laryngitis and bronchiolitis associated with beta hemolytic streptococcus group A infection	No	7.2	8
6032	4	40–45	110	55–60	M	Normal	Head and neck injuries, accident	No	7	25
1863	30	65–70	300	125–130	F	Normal	Multiple injuries, accident	No	8.8	7
1104	35	70–75	450	225–230	M	Normal	Multiple injuries, accident	No	8.4	12
1028	39	65–70	228	125–130	F	Normal	Accident	No	8.9	21
936	49.2	65–70	280	155–160	F	Normal	Liver cirrhosis	No	7.8	7
5919	63	70–75	430	185–190	M	Normal	Drowning	No	6.1	12
5828	66	60–65	320	140–150	F	Normal	Accident	No	8.2	18
5084	75	65–70	400	205–210	F	Normal	Accident	No	NA	12
5840	75.3	70–75	650	225–230	M	Hypertrophy	Ruptured abdominal aortic aneurysm	No; LV 1.6 cm, Septum 1.6 cm, RV 0.4 cm	7.8	17
5657	82.2	70–75	700	235–240	M	Hypertrophy	Natural death	No; LV 1.8 cm, Septum 1.8 cm, RV 0.2 cm	8.4	22

HW, heart weight; BW, body weight; LV left ventricle; NA, not available; RIN, RNA integrity number; RV, right ventricle; SIDS, sudden infant death syndrome; PMI, post-mortem interval.

## Data Availability

Single-cardiomyocyte WGS data is deposited in the NCBI dbGaP with accession number phs002284.v1.p1. The data are available under controlled use conditions set by human privacy regulations. Other data are available upon request.
